# Variable recombination dynamics during the emergence, transmission and ‘disarming’ of a multidrug-resistant pneumococcal clone

**DOI:** 10.1186/1741-7007-12-49

**Published:** 2014-06-23

**Authors:** Nicholas J Croucher, William P Hanage, Simon R Harris, Lesley McGee, Mark van der Linden, Herminia de Lencastre, Raquel Sá-Leão, Jae-Hoon Song, Kwan Soo Ko, Bernard Beall, Keith P Klugman, Julian Parkhill, Alexander Tomasz, Karl G Kristinsson, Stephen D Bentley

**Affiliations:** 1 Center for Communicable Disease Dynamics, Department of Epidemiology, Harvard School of Public Health, 677 Huntington Avenue, Boston MA 02115, USA; 2 Pathogen Genomics, The Wellcome Trust Sanger Institute, Wellcome Trust Genome Campus, Hinxton, Cambridge CB10 1SA, UK; 3 Department of Infectious Disease Epidemiology, Imperial College, Norfolk Place, London W2 1NY, UK; 4 Respiratory Diseases Branch, Centers for Disease Control and Prevention, Atlanta, Georgia, USA; 5 Institute for Medical Microbiology, National Reference Center for Streptococci, University Hospital, RWTH Aachen, Pauwelsstrasse 30, Aachen, Germany; 6 Laboratory of Molecular Genetics, Instituto de Tecnologia Química e Biológica, Universidade Nova de Lisboa, Oeiras, Portugal; 7 Laboratory of Microbiology, The Rockefeller University, New York, New York, USA; 8 Samsung Medical Centre, Sungkyunkwan University School of Medicine and Asia Pacific Foundation for Infectious Disease, Seoul, South Korea; 9 Department of Molecular Cell Biology, Sungkyunkwan University School of Medicine, Suwon, South Korea; 10 Hubert Department of Global Health, Rollins School of Public Health and Division of Infectious Diseases, School of Medicine, Emory University, Atlanta, Georgia, USA; 11 Centre for Respiratory Diseases and Meningitis, National Institute for Communicable Diseases, Gauteng, South Africa; 12 Clinical Microbiology Department, Landspitali University Hospital and University of Iceland, Reykjavík, Iceland; 13 Department of Medicine, University of Cambridge, Addenbrooke’s Hospital, Cambridge CB2 0SP, UK

**Keywords:** Bacterial evolution, Antibiotic resistance, Recombination, Mobile genetic elements, Coalescent analysis, Phylogeography

## Abstract

**Background:**

Pneumococcal β-lactam resistance was first detected in Iceland in the late 1980s, and subsequently peaked at almost 25% of clinical isolates in the mid-1990s largely due to the spread of the internationally-disseminated multidrug-resistant PMEN2 (or Spain^6B^-2) clone of *Streptococcus pneumoniae*.

**Results:**

Whole genome sequencing of an international collection of 189 isolates estimated that PMEN2 emerged around the late 1960s, developing resistance through multiple homologous recombinations and the acquisition of a Tn*5253*-type integrative and conjugative element (ICE). Two distinct clades entered Iceland in the 1980s, one of which had acquired a macrolide resistance cassette and was estimated to have risen sharply in its prevalence by coalescent analysis. Transmission within the island appeared to mainly emanate from Reykjavík and the Southern Peninsular, with evolution of the bacteria effectively clonal, mainly due to a prophage disrupting a gene necessary for genetic transformation in many isolates. A subsequent decline in PMEN2’s prevalence in Iceland coincided with a nationwide campaign that reduced dispensing of antibiotics to children in an attempt to limit its spread. Specific mutations causing inactivation or loss of ICE-borne resistance genes were identified from the genome sequences of isolates that reverted to drug susceptible phenotypes around this time. Phylogenetic analysis revealed some of these occurred on multiple occasions in parallel, suggesting they may have been at least temporarily advantageous. However, alteration of ‘core’ sequences associated with resistance was precluded by the absence of any substantial homologous recombination events.

**Conclusions:**

PMEN2’s clonal evolution was successful over the short-term in a limited geographical region, but its inability to alter major antigens or ‘core’ gene sequences associated with resistance may have prevented persistence over longer timespans.

## Background

Despite the high frequency of horizontal sequence transfer in the pneumococcal population, the spread of multidrug-resistant (MDR) *Streptococcus pneumoniae* has primarily reflected the global dissemination of particular clones rather than the acquisition of resistance by resident, sensitive genotypes [1]. MDR pneumococci were first identified in 1977 [2], with the phenotype becoming very common in some countries during the 1980s due to the spread of several MDR clones [3,4]. However, the absence of such bacteria from Iceland meant no penicillin resistance was detected among *S. pneumoniae* on the island until December 1988 [5]. This was in spite of the country having the highest per capita consumption of antibiotics of the Nordic countries at the time [6].

Levels of resistance subsequently rose to a peak in 1995, by which point 24.2% of Icelandic clinical pneumococcal isolates were penicillin non-susceptible [7,8]. The majority of these were of serotype 6B and also exhibited resistance to co-trimoxazole, chloramphenicol, tetracycline and macrolides [6,7,9]. Genotyping demonstrated that the pneumococci sharing these characteristics were a clonal outbreak closely related to isolates found in Spain in the late 1980s, defined as the Spain^6B^-2 or PMEN2 lineage [10], suggesting a transmission from Western Europe [6].

Consequently, an effort was initiated to reduce consumption of antibiotics [11] that achieved a 35% fall in dispensing to Icelandic children between 1992 and 1997 [12]. The prevalence of PMEN2 fell over subsequent years but despite these alterations in clinical practice and the presence of other MDR genotypes [7], serotype 6B pneumococci still constituted around 3% of Icelandic penicillin non-susceptible clinical isolates in 2010 [8]. The genotype was found to be variably prevalent in different communities, disappearing from some but persisting in others in a manner that was suggested to relate to local antibiotic prescribing rates and herd immunity [13]. Furthermore, some PMEN2 representatives with losses of particular resistances were identified, which may have been a sign of adaptation to reduced antibiotic consumption [9]. In the case of tetracycline and macrolides, reversions to susceptibility appeared to have occurred through more than one type of mutation, as they were associated with both the presence and absence of the associated resistance gene.

As well as being found throughout Western Europe from the 1980s onwards [6,14-17], the PMEN2 lineage has also been detected in the United States [18-21], South America [22,23], the Middle East [24], South-East Asia [25-27], Australia [28] and Japan [29]. Multilocus sequence typing (MLST) generally finds such isolates to be of, or similar to, sequence type (ST) 90. This suggests it is closely related to the PMEN22 lineage, first identified in countries around the Mediterranean [30,31] and subsequently found in Western Europe and the United States [32,33]. The PMEN22 genotype is associated with ST273, which differs from ST90 at only two of the seven MLST loci, and generally has a similar resistance profile to PMEN2 with the exception that representatives are typically β-lactam susceptible. An international collection of 189 isolates was, therefore, assembled to identify the relationship between these two lineages and trace the emergence and spread of the PMEN2 clone. This included 118 isolates from Iceland to reconstruct the outbreak on this island in detail, and thereby characterise its initial success and subsequent population dynamics in response to altered antibiotic dispensing practices.

## Results

### Multiple resistance elements across a diverse collection

**Figure 1:**
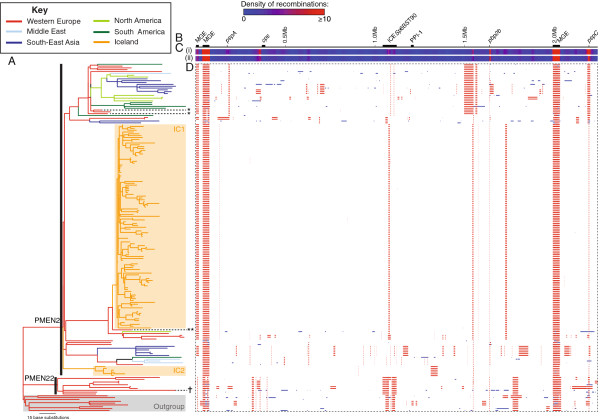
**Phylogenomic analysis of the sequenced isolates. (A)** Maximum likelihood phylogeny of the isolates based on likely point mutations. The branches of the phylogeny are coloured according to the geographical location of the isolates, as reconstructed through the tree on the basis of maximum parsimony. The PMEN2 and PMEN22 clades are indicated by the black vertical bars; the grey shaded box marks the ‘Outgroup’ clade. Isolates from Iceland are indicated by the orange boxes highlighting clades IC1 and IC2. Isolates from Spain previously providing evidence of transmission from continental Europe are marked with single asterisks; the German isolate most closely related to clade IC1 is marked with a double asterisk. The PMEN22 isolate that has become resistant to β-lactams is marked by the † symbol. **(B)** Simplified annotation of the reference genome against which sequence reads were mapped. The positions of putative mobile genetic elements (MGEs) is marked, along with the capsule polysaccharide synthesis locus (*cps*), Pneumococcal Pathogenicity Island 1 (PPI-1), the gene for penicillin-binding protein 2B (*pbp2b*), and the antigen-encoding genes *pspA* and *pspC*. **(C)** Density of recombination events across the genome: (i) across the entire collection, (ii) within only the PMEN2 clade. **(D)** Distribution of recombination events. The dashed line defines a panel consisting of a row for each of the sequenced isolates in the collection, with a column for each base in the reference sequence. Putative recombination events are indicated by the coloured blocks: red events are reconstructed as occurring on internal branches and are, therefore, shared by multiple isolates through common descent, whereas blue blocks are recombinations occurring on terminal branches, which are unique to individual isolates.

A total of 189 isolates from twelve countries that spanned the period from 1988 to 2009 were sequenced as multiplexed libraries using the Illumina platform [see Additional file 1: Table S1]. Reads were mapped against the reference genome of *S. pneumoniae* 670-6B and bases called using previously-defined criteria [34], leading to the identification of 36,038 polymorphic sites. Reconstructing the evolutionary history of the lineage as described previously [35] suggested that 63,053 base substitutions, primarily the result of 616 recombinations, had occurred during the divergence of the isolates in the collection (Figure 1). Analysis with an alternative algorithm for detecting recombination produced similar results [see Additional file 2: Figure S1]. The reconstruction involved 3,955 point mutations, giving an estimate of the overall per site *r/m* (the ratio of base substitutions introduced by recombination relative to point mutations) of 14.9; this compares with a typical range from around 0.1 to more than 30 for common pneumococcal lineages [36,37]. However, it is clear that many of these events occurred within putative mobile genetic elements (MGEs) and were, therefore, unlikely to be the products of genetic transformation, but rather seemed to primarily represent the consequences of phage movement. Excluding the 242 recombinations within MGEs reduced the *r/m* due to homologous recombination to 9.80. This subset of recombinations had a mean length of 8.8 kb and followed an approximately exponential length distribution [see Additional file 3: Figure S2] [38], with each event importing a mean of 104 base substitutions. The import of sequence via transformation appears to have happened most frequently at the penicillin-binding protein genes *pbp2x* (just upstream of the capsule polysaccharide synthesis, or *cps*, locus) and *pbp2b*, which are important in determining resistance to β-lactams, as well as around the *pspC* gene, which encodes the Pneumococcal Surface Protein C antigen [39]. No putative recombinations were found to affect the *cps* locus, the gene cluster that determines an isolate’s serotype. However, this did not preclude a switch from serotype 6B to 6A occurring in isolate 1014–00 through the A584G base substitution in *wciP* causing an N195S amino acid change in the corresponding rhamnosyl transferase enzyme, a polymorphism previously associated with determining which of these two polysaccharide types an isolate produces [40]. Only one other base substitution, a non-synonymous change within the regulatory gene *wze*, occurred within the *cps* locus on the same branch of the phylogeny, suggesting this alteration could have occurred either through recombinations importing limited diversity or through point mutations.

**Figure 2:**
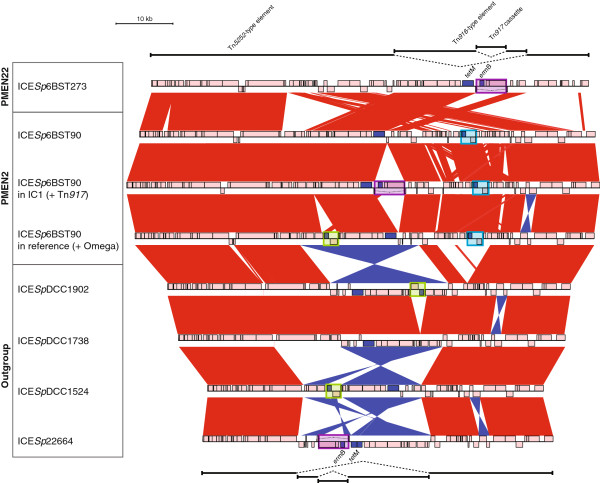
**Comparison of integrative and conjugative elements (ICE) identified in the collection.** Eight examples of composite Tn*5253*-type ICE found in the collection are aligned: ICE*Sp*6BST273, found in PMEN22, ICE*Sp*6BST90, found in PMEN2 (examples modified by the integration of macrolide resistance cassettes are included), and four ICE representing the diversity found in the outgroup. Red bands indicate BLAT matches between sequences in the same orientation and blue twisted bands indicate BLAT matches between sequences in opposite orientations. In both cases, the intensity of the colour indicates the strength of the match. Protein coding sequences (CDSs) are indicated by pink boxes, except those encoding antibiotic resistance determinants, which are coloured dark blue. The vertical position above or below the central line indicates whether the CDS is encoded on the forward or reverse strand of the sequence. The *tetM* tetracycline resistance gene, found within the Tn*916*-type elements, is marked at the top and bottom of the alignment. The *ermB* macrolide resistance gene is found within Tn*917* cassettes (boxed in purple throughout the alignment) or Omega cassettes (boxed in green throughout the alignment). The *cat* chloramphenicol acetyltransferase is carried on the linearized pC194 plasmid, indicated by blue boxes on the annotation. BLAT, BLAST-like alignment tool.

The resultant phylogeny robustly separated the PMEN2 and PMEN22 clones from a third, distinct clade [see Additional file 4: Figure S3]. All three groups showed evidence of having acquired Tn*5253*-type integrative and conjugative elements (ICE; Figure 2). These are large composite elements in which a Tn*916*-type element harbouring the tetracycline resistance gene *tetM* is inserted into a larger Tn*5252*-type element [41,42]. The oldest and most diverse clade, labeled ‘Outgroup’ in Figure 1, was composed of isolates that were universally β-lactam sensitive. The ICE within this Outgroup clade could be represented by four archetypes, which exhibited similarity in gene content but a large degree of structural variation, such as inversion of Tn*916*-type components, differences in the length of the Tn*5252*-type element and variation in the presence of different macrolide resistance cassettes within the Tn*916*-type element (Figure 2 and Additional file 5: Figure S4) [35]. This made it difficult to deduce whether these represent the entry of a single element that has been maintained (albeit with *in situ* modification) across the clade, or multiple acquisitions of related ICE.

A second clade corresponded to the PMEN22 lineage (Figure 1). This clade had a relatively high *r/m*: 18.84 overall and 13.73 excluding recombinations within MGEs. These isolates all inherited the insertion of a single, novel large Tn*5253*-type ICE, consequently named ICE*Sp*6BST273 (Figure 2 and Additional file 6: Figure S5). Macrolide resistance across the clade was mainly the result of a Tn*917* cassette [43] within the ICE carrying a ribosomal RNA methylase gene (*ermB*). Chloramphenicol resistance was the result of a *cat* chloramphenicol acetyltransferase within a pC194-type sequence [44] as part of a gene cluster very similar to the Ω*cat*(pC194) element found within Tn*5253*[45] [see Additional file 6: Figure S5]. *De novo* assemblies indicated this sequence could transpose to different loci within the genome, sometimes integrated into the ICE or Pneumococcal Pathogenicity Island-1, as was observed for the archetypal element [45]. This represents a further example of sequence exchange between ICE and this island [42,46]. The PMEN22 clade was generally β-lactam sensitive, except for a single resistant isolate (SN33007) that was predicted to have imported 23 segments of sequence, totaling just under 254 kb in length, that affected the penicillin binding protein genes *pbp1a, pbp2x* and *pbp2b*, but not *pbp2a* or *murMN*.

### Diversification and dissemination of PMEN2

β-lactam resistance was independently acquired by the largest clade, corresponding to PMEN2, all isolates of which were distinguished from the Outgroup and PMEN22 clades by 17 shared segments of recombinant sequence that totaled 156 kb in length. This resulted in the import of resistance-associated alleles of *pbp1a*, *pbp2x* and *pbp2b*[47], but no contemporaneous recombinations affecting *pbp2a* or *murMN* were detected. Resistance to tetracycline and chloramphenicol resulted from the acquisition of ICE*Sp*6BST90 on the same branch of the phylogeny, which was shared by all PMEN2 representatives through common descent [see Additional file 6: Figure S5]; this contained both a Tn*916*-type element and a Ω*cat*(pC194)-like element [44,45] [see Additional file 7: Figure S6]. Therefore, the antibiotic resistance determinants of PMEN2 and PMEN22 appear to have been entirely independently acquired. Many isolates were also resistant to macrolides owing to the acquisition of the *ermB* gene carried on either the Omega cassette [35] or Tn*917*, both of which appear to have been acquired more than once during the evolution of PMEN2 [see Additional file 5: Figure S4]. Based on one atypical Tn*916*-type sequence [see Additional file 8: Figure S7], it seems likely that both cassettes were acquired by a recent common ancestor of one particular clade of three isolates, thereby arranging two highly similar *ermB* genes in tandem. A subsequent intragenomic recombination between them appears to have deleted the intervening sequence, resulting in a reversion to tetracycline susceptibility owing to the loss of the *tetM* gene, but leaving the aminoglycoside resistance gene *aph3’* and *ermB* in its place.

A plot of the root-to-tip distance of the isolates within the PMEN2 clade relative to their date of isolation revealed a significant linear correlation (*n* = 168, *R*^2^ = 0.63, *P* value <2.2 × 10^-16^) that suggested a date of origin around 1967 [see Additional file 9: Figure S8A]. A Bayesian coalescent analysis of the clade estimated the most likely date of origin to be 1969 (95% credibility interval, 1962 to 1974), implying a point mutation rate of 1.11 × 10^-6^ substitutions per site per year (95% credibility interval of 9.82 × 10^-7^ to 1.24 × 10^-6^ substitutions per site per year). This origin is likely to have occurred in Western Europe (Figure 1), in accordance with the site of the first identification of the clone [17]. PMEN2 then seems to have spread to other continents on multiple occasions, concurrent with isolates undergoing extensive diversification across much of the genome.

### Clonal evolution within Iceland

By contrast, the Icelandic isolates formed two clades (IC1 and IC2), neither of which exhibited strong evidence of having engaged in homologous recombination (Figure 1). Clade IC2 appears to represent a relatively unsuccessful transmission to the island; all six representatives were macrolide sensitive, as they carried versions of ICE*Sp*6BST90 lacking an *ermB*-containing cassette. The most recent common ancestor of IC2 was estimated to have existed around 1984 (95% credibility interval, 1980 to 1987) shortly before the first clinical isolate was detected, but no isolates belonging to the clade were detected from 1994 onwards. The IC2 isolates corresponded to three similar pulsed field gel electrophoresis (PFGE) patterns [9]; although no putative homologous recombinations were identified within this group that might have caused such diversification, there were five recombination events within MGEs indicating that the acquisition of autonomously mobile elements may have caused the alterations. The larger IC1 clade was composed of 112 isolates and appears to have entered Iceland at a similar time, around 1986 (95% credibility interval, 1985 to 1987). These isolates were generally macrolide resistant due to the insertion of Tn*917* into ICE*Sp*6BST90 (Figure 2).

Only 20 recombinations were detected within clade IC1, of which 14 occurred within prophage and appear to represent the movement of viral sequences. Each of the remaining six events imported no more than eight substitutions, far below the mean of 104 substitutions per recombination in the wider collection. Consequently, all of the isolates tested near the start of the outbreak corresponded to a single PFGE type [9], and the clade’s *r/m* was 0.18, falling to 0.024 when recombinations within MGEs were excluded. This seems largely due to the insertion of a prophage, henceforth referred to as ΦIC1, into the *comYC* (also known as *comGC*) gene, which encodes a component of the competence pilus essential for the uptake of exogenous DNA into the cell [48]. The integration of prophage into this locus has previously been experimentally associated with the loss of competence for transformation [35]. Sequence read mapping to ΦIC1 revealed that related viruses with very similar integrases, which seem to target the same locus based on cases where the prophage could be assembled *de novo*, appear to have inserted into PMEN2 representatives across the world on several occasions [see Additional file 10: Figure S9 and Additional file 11: Figure S10]. This analysis of prophage distribution also showed that the ΦIC1 virus is absent from the chromosomes of many isolates within clade IC1, indicative of frequent loss. Based on *de novo* assemblies, the *comYC* gene seems to have returned to a functional form in these samples, demonstrating that the MGE’s excision is precise. However, loss of the prophage was not associated with a detectably elevated rate of sequence import, as only one of the putative homologous recombination events was associated with isolates carrying ‘repaired’ *comYC* genes.

### Phylodynamics of the Icelandic outbreak

The phylogeny supported the hypothesis that PMEN2 entered Iceland from Western Europe [6] (Figure 1 and Additional file 4: Figure S3), but not the suggestion of Spain in particular being the source, which was based on genotyping of isolates SP522 and SP681 (Figure 1). The isolate most closely related to clade IC1 was from Germany, albeit from 1998, post-dating the estimated dates of clades IC1 and IC2 arriving in Iceland by around a decade. The transmission and population dynamics of clade IC1 subsequent to its entry into Iceland were reconstructed using a coalescent model to combine the sequence alignment with the detailed epidemiological information available. This analysis could be performed independently of the rest of the collection, as this clade alone showed a significant correlation between root-to-tip distance within the clade and day of isolation (*n* = 112, *R*^2^ = 0.56, *P* value <2.2 × 10^-16^; Additional file 9: Figure S8B). This estimated the date of IC1’s common ancestor as around October 1987 (95% confidence interval September 1986 to August 1988), consistent with the overall analysis of the PMEN2 clade.

**Figure 3:**
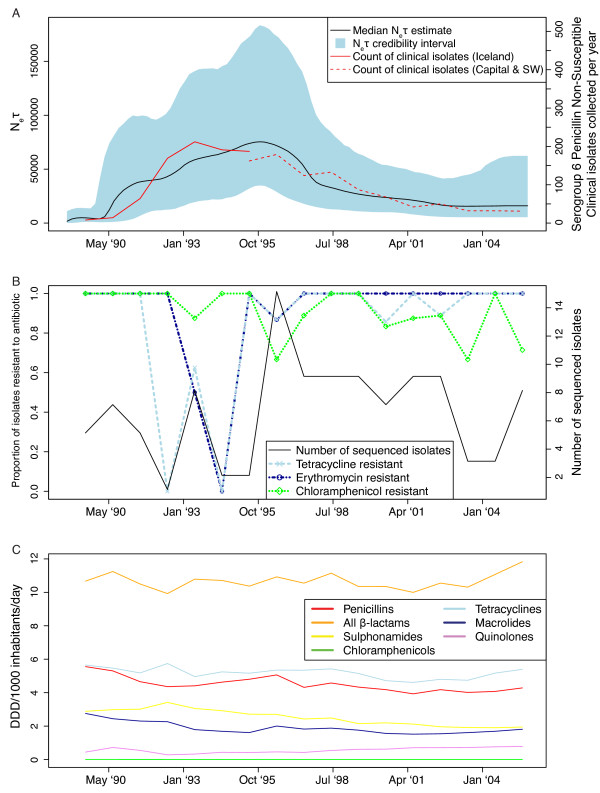
**Temporal dynamics of clade IC1 in Iceland. (A)** Bayesian skyline plot of the product of effective population size and generation time over the course of the outbreak. The median estimate of this parameter is displayed as the black line, while the 95% credibility interval is indicated by the blue filled area. The solid red line indicates the recorded numbers of penicillin non-susceptible pneumococci of serotype 6B collected from cases of disease in Iceland between 1989 and 1995. The dashed red line indicates the available figures for the number of clinical isolates meeting these criteria collected from the Capital and Southwestern areas from 1995 onwards, which account for more than 90% of the penicillin non-susceptible pneumococci identified in Iceland [8]. **(B)** Proportion of clade IC1 isolates resistant to tetracycline, erythromycin and chloramphenicol over time. The total number of sequenced isolates from clade IC1 is represented by the black line, relative to the right side vertical axis. The proportions of these isolates resistant to selected antibiotics in each year, excluding those for which no phenotype was available or that were defined as having an ‘intermediate’ resistance phenotype [see Additional file 1: Table S1], are represented by the coloured lines relative to the left side vertical axis. **(C)** Use of antibiotics in Iceland. Annual defined daily doses (DDD) in Iceland for classes of antibiotics against which IC1 isolates were found to have resistance are displayed. The data in panels **(B)** and **(C)** are plotted using the midpoint of each year such that they are displayed on approximately the same timescale as the changes in the effective population size of IC1 in panel **(A)**.

The change in the clade’s prevalence was estimated using a Bayesian skyline plot (Figure 3) [49]. This traces changes in the product of the effective population size (*N*_e_) and the generation time (τ); assuming τ to be constant, the plot can be taken as an estimate of *N*_e_ over time. This analysis found *N*_e_ to start at zero when the genotype entered the country, rising to a peak in the mid-1990s, before dropping to a much lower level by 2004. Comparison with the recorded numbers of penicillin non-susceptible serogroup 6 isolates recovered from disease over the same period revealed a similar pattern of rise and fall. There was not a clear relationship with the overall levels of usage of any one particular antibiotic over time, although post-1995 there was a consistent fall in the usage of penicillins, sulphonamides and macrolides. However, these chart the overall consumption of antibiotics, whereas the reduction in prescribing was targeted at children (the primary hosts for the pneumococcus), in whom consumption fell by 35% [12]. Furthermore, it may well be that these overall trends masked more obvious changes at the level of individual communities, as seen by location-specific epidemiological surveillance [13].

**Figure 4:**
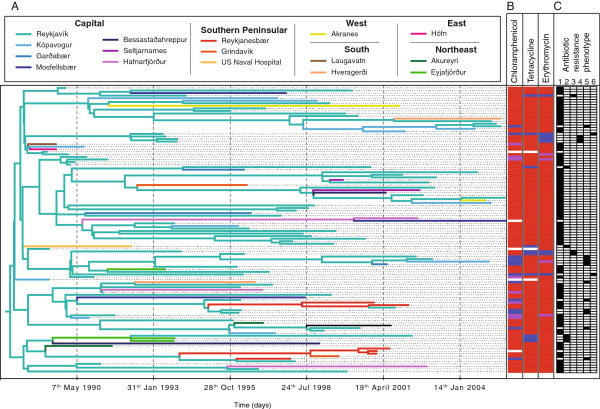
**Transmission of clade IC1 within Iceland. (A)** A phylodynamic analysis was conducted to reconstruct the transmission of the IC1 clade across Iceland. The tree is coloured according to the most likely location of each branch, as determined through the discrete states phylogeographic model described by [60]. The locations are clustered by region in the key. **(B)** Each isolate’s resistance profile is represented by red bars indicating full resistance, purple bars indicating intermediate levels of resistance and blue bars indicating sensitivity to the antibiotics annotated at the top of the column; white bars indicate missing data. **(C)** The isolates are classified according to the phenotypic groups one to six defined in [9] based on resistance phenotype and genotype, which are described in the main text.

To trace transmission at a more local level, a discrete state phylogeographic model was fitted as part of the coalescent analysis (Figure 4). This analysis indicated Reykjavík was an important source of transmissions, being the reconstructed location of more than 90% of the phylogeny’s internal nodes, including the root node. However, this might have been anticipated from the composition of the collection, given that 81% of the IC1 isolates were from the capital and surrounding suburbs, which contained around 57% of the total Icelandic population at the start of the outbreak [6]. Correspondingly, randomly permuting the locations of the isolates 100 times found the same, or greater, proportion of internal nodes to be ascribed to Reykjavík in 88 cases, with every permutation attributing the root node as emanating from the capital. By contrast, the second most common location of Reykjanesbær was associated with five internal nodes in the original analysis; a similar pattern was only reconstructed in a single permutation. Hence, the phylogeographic analysis provided significant evidence of a series of transmissions across the relatively densely populated southern peninsular of the island, distinct from the network of transmissions involving Reykjavík.

### ‘Disarming’ of an MDR clone

**Figure 5:**
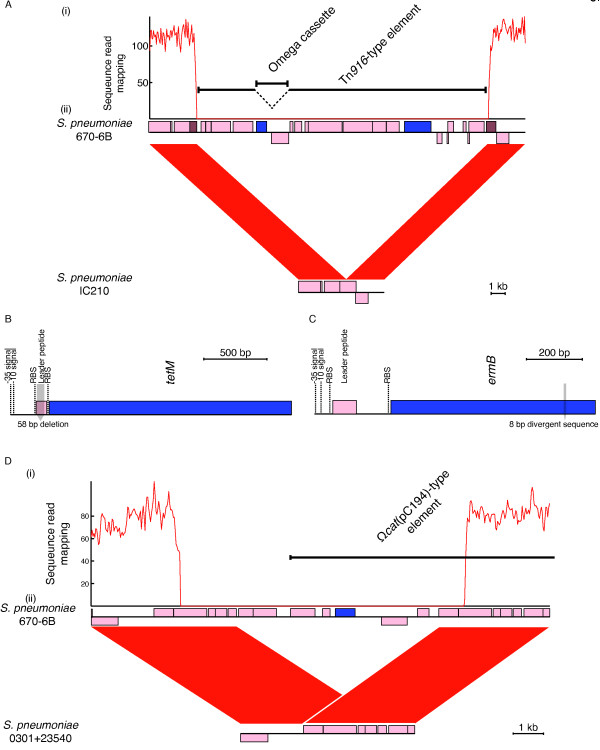
**Loss of resistance within clade IC1. (A)** Loss of erythromycin and tetracycline resistance in group 3 isolates. (i) Plot of coverage calculated by mapping sequence reads from group 3 isolate IC210 to the reference genome Tn*916*-type element, showing no coverage across the transposon. This suggests it has been lost from the genome. (ii) Comparison of *de novo* assembly of isolate IC210 with the reference sequence. The red bands between the two sequences indicate BLAT matches between sequences in the same orientation. The annotation of the reference sequence is at the top; protein coding sequences (CDSs) are indicated by pink boxes, except for those that encode resistance determinants, which are coloured blue. The brown boxes indicate the fragments of a CDS that is reformed by the apparent excision of the transposon, as seen in the assembled sequence of isolate IC210 at the bottom of the diagram. **(B)** Loss of tetracycline resistance in group 2 isolates. The figure shows the structure of the *tetM* gene, including the positions of the promoter elements, leader peptide and ribosome binding sites (RBS). A 58 bp deletion within the leader peptide, at the bases indicated by the shaded bar, appears to result in the inactivation of the gene. **(C)** Loss of erythromycin resistance in group 4 isolates. The structure of the *ermB* gene is displayed as described for (B), with the position of the sequence alteration leading to a frameshift mutation indicated by the shaded bar. **(D)** Loss of chloramphenicol resistance within some group 5 isolates through deletion of the *cat* chloramphenicol acetyltransferase gene on the Ω*cat*(pC194) element. (i) Plot of mapped sequence read coverage and (ii) comparison of *de novo* assembly with the reference genome for group 5 isolate 0301 + 23540, displayed as in (A). BLAT, BLAST-like alignment tool.

All isolates prior to 1992 for which definitive phenotypes were available were resistant to tetracycline, macrolides and chloramphenicol [see Additional file 1: Table S1 and Figure 3B]; however, after the reduction in antibiotic dispensing, isolates that appeared to have reverted to a susceptible phenotype were observed. Based on isolates’ resistance profile and the presence of the *tetM* and *ermB* genes, six groups were identified (Figure 4). Group 1 isolates had the resistance profile expected from the original genotype, whereas deviations were classed in groups 2 to 6 [9]. Group 2 isolates were tetracycline sensitive, despite a DNA probe indicating that the *tetM* gene was present. Five examples were evident in this collection, distributed polyphyletically as one clade of three isolates and two singletons. In each case, the alteration in phenotype was associated with the same 58 bp deletion upstream of *tetM* (Figure 5). Normally, *tetM* transcription is thought to be repressed through an attenuation mechanism: the rapid initiation of translation of a leader peptide results in the formation of a hairpin loop that terminates RNA synthesis before the resistance determinant is transcribed [50]. However, in the presence of tetracycline, the translation of the leader peptide is delayed enough that a different set of stem-loops form, resulting in the expression of the *tetM* coding sequence. Therefore, it seems likely that the 58 bp deletion disrupted this mechanism and thereby constitutively inhibited the synthesis of the TetM protein. This mutation was not observed in any non-Icelandic isolates.

Group 3 isolates were again tetracycline sensitive, but differed in also being susceptible to macrolides, having apparently lost both the *tetM* and *ermB* genes. This phenotype appears to have emerged twice within clade IC1; in both cases, it was associated with the excision of the Tn*916*-type element. This loss was evident both from comparison of *de novo* assemblies with the reference, as well as an absence of sequence read mapping to the element (Figure 5). The removal of the ICE was precise and led to the restoration of a gene encoding a zinc-dependent peptidase that appeared to have been split by the insertion of the Tn*916*-type component in the formation of the composite ICE. Aside from the deletion within Tn*916* driven by the tandem *ermB* genes, this element was stable across the rest of the PMEN2 clade, as observed in other lineages [35].

Group 4 isolates were tetracycline resistant but macrolide sensitive, despite retaining the *ermB* gene and, by implication, the Tn*916*-type element. This phenotype was found in a single clade of three isolates within IC1. *De novo* assembly revealed this to be the consequence of 8 bp of the *ermB* CDS being replaced by 7 bp of divergent sequence, introducing a frameshift mutation that truncated the resistance gene. The disruptive 7 bp sequence is an inverted copy of the same pattern of bases found downstream within *ermB*, suggesting that the change may have been driven by an unusual intragenomic recombination. This mutation only appears to have occurred once across the entire collection.

Group 5 isolates were defined as those displaying susceptibility to chloramphenicol; however, it was not previously possible to determine whether this was correlated with the loss of the *cat* chloramphenicol acetyltransferase gene. Analysis of the *de novo* genome assemblies revealed that the frequent emergence of this phenotype in the collection was, once more, associated with isolates both possessing, and having lost, the ICE-encoded gene. One clade of isolates had a 9.36 kb deletion within the Tn*5252*-type component of the ICE that eliminated part of the Ω*cat*(pC194)-like element, including the *cat* gene; this appears to have resulted from an intragenomic recombination between the long tandemly-repeated sequences on either side (Figure 5D). This unstable element also appears to have been lost outside of Iceland on several occasions [see Additional file 6: Figure S5]. However, no obvious candidate mutations causing chloramphenicol susceptibility could be found affecting the Ω*cat*(pC194)-like element in other group 5 representatives, although ambiguities in establishing the element’s mobility and insertion site made it difficult to fully characterize in all isolates. Group 6 isolates, susceptible to chloramphenicol, tetracycline and macrolides despite retaining the required genes, similarly lacked any obvious mutation underlying their resistance profile.

## Discussion

The PMEN2 lineage appears to have emerged in the late 1960s, around the same time as PMEN1 (Spain^23F^-1), when β-lactam non-susceptible pneumococci were first being detected [51]. Therefore, PMEN2 is likely to have been one of the first such genotypes. Both PMEN1 and PMEN2 seem to have acquired near-identical penicillin-binding protein sequences, and both acquired *cat* from pC194, and *tetM* from Tn*916*, as part of a Tn*5253*-type element [42,47]. While PMEN1 was more successful in many countries, it was PMEN2 that predominated in Iceland, despite the presence of other MDR clones (PMEN1 among them) [7]. More specifically, clade IC1 of PMEN2 appears to have been much more successful than IC2, which may reflect the macrolide resistance cassette carried by clade IC1. This is supported by the observation that treatment with erythromycin was associated with double the risk of carrying penicillin resistant pneumococci as treatment with β-lactams in Iceland at the time [52].

The fall in dispensing of antibiotics to children that started in 1992 is likely to have reduced the selection pressure driving PMEN2’s success within Iceland [53]. In the mid-1990s, this seems to be reflected in the instances of the MDR clone being ‘disarmed’. Each of the reversions to chloramphenicol, tetracycline and macrolide sensitivity occurred both in the presence and absence of the relevant resistance gene; furthermore, some of the specific mutations were homoplasic within clade IC1. Such convergent evolution is likely driven by selection; this would suggest that these resistance mechanisms impose a fitness cost upon the host cell and, therefore, tend to degrade as they became less advantageous [9]. However, these variants appear only to have enjoyed limited success, and primarily seem to have been observed when the PMEN2 clone was present at a comparatively high frequency relative to the use of antibiotics in children selecting for resistance. Both the count of clinical isolates and apparent change in *N*_e_τ indicate a drop in the prevalence of clade IC1 beginning in the mid- to late-1990s. Once the clone fell to a lower, stable frequency in response to this change in prescribing, the pressure to revert to a sensitive phenotype is likely to have been reduced. This may explain why no further reversions to macrolide resistance were observed from 1997 onwards. Alternatively, the limited success of these variants may indicate that such mutations are only adaptive over the short-term to atypically low antibiotic use in a certain community [9].

By contrast, non-ICE ‘core’ sequences that caused antibiotic resistance were stable within clade IC1. For instance, resistance to penicillins and co-trimoxazole was retained despite the declining use of these drugs. This is inevitable in the context of the clonal evolution of clade IC1; in the absence of allelic replacement at *dyr* (encoding dihydrofolate reductase, the sequence of which determines susceptibility to trimethoprim)*, folP* (encoding dihydropteroate synthase, the sequence of which determines susceptibility to sulphonamides) or penicillin-binding protein genes, reversion to the sensitive ancestral sequences was not possible. This is similar to the pattern of evolution observed in serotype 3 isolates [37] and in contrast to the rapid import of divergent sequence observed in PMEN1 [35] and PMEN2 isolates from outside Iceland in this collection. This absence of transformation largely appears to be the consequence of the disruption of *comYC* by the insertion of prophage ΦIC1, although it is not clear why the isolates lacking this element still do not import sequence diversity from other pneumococci. It may be that the prophage is unstable only during disease or laboratory culturing, and that, in fact, the prophage remained inserted in all carried IC1 isolates. This failure to adapt to changing levels of antibiotic use in children through horizontal import of sequence may have played a part in the subsequent decline of clade IC1 in Iceland after the mid-1990s.

Alternatively, the lack of antigenic diversification resulting from the absence of recombination may have contributed to the genotype’s fall in prevalence. The high density of recombination events affecting the *pspC* locus outside of the IC1 clade suggests a selective pressure to alter such antigen-encoding genes. Therefore, the decrease in clade IC1’s frequency may be the consequence of the resident population accumulating herd immunity to the genotype [13]; this may be particularly acute in Iceland, as the phylogeography of the lineage suggests that only Reykjavík and the Southern Peninsular region sustained the genotype long-term, potentially acting as a source for the rest of the country. Declining transmission within these communities may have resulted in an ensuing fall in the rate of spread of the clade to other locations. Furthermore, as the acquisition of a capsule differing extensively from 6B seems unlikely within clade IC1, it seems destined for elimination following the introduction of polysaccharide conjugate vaccines (which induces immunity against both serotype 6A and 6B) in 2011 [8].

## Conclusions

PMEN2 emerged in the 1960s, and a single transmission into Iceland in the late 1980s appears to have been particularly successful despite its lack of sequence import through homologous recombination. While this limitation did not appear to inhibit the loss of antibiotic resistance found on mobile genetic elements, it prevented modification of ‘core’ sequences associated with resistance and antigenic diversification, which likely contributed to its decline after the mid-1990s.

## Methods

### Selection of isolates for sequencing

Isolates with an MLST profile similar to that of ST90 were supplied by the sources listed in Additional file 1: Table S1. In the case of the Icelandic representatives of PMEN2, isolates were selected to produce a temporally even sample over the course of the lineage’s spread across the island when possible; these were supplemented by a set specifically chosen as exhibiting atypical resistance profiles. Isolates were supplied by the same set of clinical centres over the course of the study, but the region from which they received samples was reduced from the entire island pre-1995 to only the Capitol and Southwestern regions post-1995. However, these two districts accounted for the vast majority of isolates across all years.

### DNA sequencing and phylogenomics

DNA was sequenced as multiplexed libraries on the Illumina Genome Analyzer II and HiSeq platforms, and the raw data deposited in the European Nucleotide Archive (ENA), as described by the read lengths and accession codes in Additional file 1: Table S1. Serotype and sequence type were identified as described in [35] to check the integrity of sample handling. Sequence reads were mapped against the reference sequence of *S. pneumoniae* 670-6B (EMBL accession code: CP002176), an annotated complete genome [54], using SMALT v0.6.4 as described previously [38]. MGEs were identified in the reference genome through alignments with other complete pneumococcal genomes. Polymorphisms were identified using criteria defined in [34]. Samples showing signs of contamination, based on the quality of *de novo* assemblies and signs of the presence of multiple alleles in mapped sequence read data, were discarded. Only samples with a mean coverage across the entire reference genome above 25 fold and able to call bases at >90% of reference positions, were used in the phylogenomic analysis. This generated a reference-based whole genome alignment for phylogenetic and clustering analyses. The identification of putative recombination events, and generation of a phylogeny based on vertically-inherited point mutations, was performed as described previously [35]. This method identifies recombinations as regions of the genome affected by high densities of base substitutions on individual branches of the phylogeny, which may reflect homologous recombination or the movement of MGEs. Therefore, to exclude horizontal sequence exchange likely driven by the latter mechanism, homologous recombination events were defined as those occurring outside of the annotated MGEs in the reference sequence.

Independently, the same whole genome alignment was analysed using BRATNextGen [55], assuming three clusters, using a learned value of alpha, a window size of one kilobase and a significance threshold *P* value of 0.05 (as calculated from 100 permutations).

### Bayesian coalescent analyses

For the analysis of the PMEN2 clade, the topology of the phylogeny was fixed as that of the relevant clade of the overall phylogeny, in order to maintain consistency with the prediction of recombinations. Analysis with Path-O-Gen [56] revealed that this phylogeny displayed a significant correlation between the root-to-tip distance of a sample and its year of isolation [see Additional file 9: Figure S8A]. The nucleotide alignment used was the subset of positions at which base substitutions occurred within the clade, with those substitutions introduced by recombination excluded from the alignment, and the dates of isolation used were the year, or range of years, provided in Additional file 1: Table S1. Samples for which no information regarding date of isolation was available were each associated with a uniform prior distribution across the range of years of isolation of the rest of the collection. Analysis with Bayesian evolutionary analysis using sampling trees (BEAST) [57] used a general time-reversible (GTR) substitution model with a single rate category and a lognormal relaxed molecular clock rate [58]. A Bayesian skyline plot was used as the population demography prior [49]. Ten chains of 100 million states each were run; 50 million generations were removed as burn-in and the remaining data combined. All parameters were estimated with estimated sample size (ESS) values above 200, with the exception of the year of isolation of samples SPN11926 and SN11927. However, joint marginal plots demonstrated that neither of these parameters showed a significant correlation with any of the values described in the text. This model was compared to four others: a strict molecular clock, a random molecular clock, a lognormal relaxed clock with four rate categories and a lognormal relaxed clock with a Hasegawa, Kishino and Yano (HKY) substitution model. These analyses used the same input data and were processed in the same way, and the same parameters were estimated with ESS values above 200. As assessed by Bayes factors [59], these alternative models all fitted the data less well than that used for the analysis described in the text [see Additional file 12: Table S2]. In the case of the model using four rate categories the fit was only slightly worse as judged by Bayes factors. However, the coefficient of variation in the absence of multiple rate categories (median estimate of 0.42; 95% credibility interval of 0.32 to 0.52) was not found to provide evidence of extensive rate heterogeneity, hence this alternative model was rejected.

For the analysis of the IC1 clade, the day of isolation was used, where available; otherwise, the 15th day of the month of isolation was used as an approximation of the day of isolation. Again, a significant correlation between root-to-tip distance and day of isolation was observed [see Additional file 9: Figure S8B]. The analysis was otherwise conducted as described for the overall PMEN2 clade. The displayed tree and Bayesian skyline plot was calculated using 10,000 trees (matched to the equivalent number of states) derived through removing 50 million generations from each of the ten 100 million generation chains as burn-in, then resampling the remaining generations at a frequency of 1 per 50,000. All values were estimated with an ESS greater than 200. This model was also compared to the same four alternative models as described above [see Additional file 13: Table S3], and once more the only other model to give a similarly good fit to the data as the described analysis was that using a lognormal relaxed molecular clock with four rate categories. However, the inclusion of the rate categories did not significantly improve the fit, and the coefficient of variation in the described analysis with a single rate category had a median value of 0.33 (95% credibility interval of 0.22 to 0.45), which suggested there was not strong evidence of extensive rate heterogeneity. Hence, the model with multiple rate categories was rejected on the grounds of parsimony.

A discrete state phylogeographic model was used to reconstruct the transmission of the IC1 clade across Iceland [60]. When testing for significance of the observed patterns through permutations, 100 versions of the alignment were generated in which the locations were randomly reassigned to isolates. The tree, sequences and dates of isolation were held constant. Each permutation was analysed with BEAST using the same model, but with a chain length of 10 million generations; this was sufficient for the parameters of the discrete states phylogeographic model to have converged. One million of these were removed as burn-in and the remaining trees (sampled at a frequency of 1 per 5,000 generations) used to determine the robustness of the phylogeographic reconstruction.

### Analysis of accessory genome distribution

For identification of novel accessory genome components, Illumina sequence reads were assembled using Velvet [61]. This was run iteratively to identify the k-mer that gave the highest N_50_ value, while maintaining an expected level of coverage above 20, as described previously [35]. Scaffolds were then generated using SSPACE2 [62], which were subsequently improved using the post assembly genome improvement toolkit (PAGIT) pipeline [63]. Sequence alignments were performed using BLAT [64] with default settings and displayed using ACT [65]. The ICE displayed in Figure 2 have been submitted to the ENA with the following accession codes: ICE*Sp*22664 (HG799489), ICE*Sp*DCC1902 (HG799491), ICE*Sp*DCC1738 (HG799492), ICE*Sp*DCC1524 (HG799493) and ICE*Sp*6BST273 (HG799495). ICE*Sp*SPN8332, displayed in Additional file 8: Figure S7, has been submitted to the ENA with accession code HG799498. The prophage sequence in Additional file 11: Figure S10 have been submitted to the ENA with the following accession codes: ϕDCC1738 (HG799497), ϕK13-0810 (HG799496) and ϕIC1 (HG799490).

To ascertain the distribution of sequence reads across the collection, reads were mapped to the accessory genome component using BWA v0.7.3 [66]. The coverage plots were then generated using Samtools [67]. Heatmaps were generated using Biopython [68].

## Competing interests

WPH has consulted for GlaxoSmithKline. KPK has consulted for Pfizer, GlaxoSmithKline and Merck. KGK has an investigator-initiated study sponsored by GlaxoSmithKline. SDB has consulted for Merck. The other authors declare that they have no competing interests.

## Authors’ contributions

SDB, AT, JP, KGK and NJC conceived and designed the study; NJC, SRH and KGK analysed the data. KGK, AT, WPH, LM, ML, HL, RSL, JHS, KSK, BB and KPK collected and summarised data for the study. All authors drafted and approved the final manuscript.

## Supplementary Material

Additional file 1: Table S1 Information regarding the source of isolates, their phenotypic properties, and the accession codes for the raw sequence data used in this analysis. Resistance to antibiotics is detailed where the information is available; numerical values indicate a minimum inhibitory concentration in milligrams per litre, whereas the outcome of less precise phenotypic tests is indicated by the terms ‘sensitive’, ‘intermediate’ or ‘resistant’. Click here for file

Additional file 2: Figure S1 Analysis of the whole genome alignment using BRATNextGen. (A) Maximum likelihood phylogeny, as displayed in Figure 1. (B) Simplified annotation of the reference genome as displayed in Figure 1. (C) Results of the BRATNextGen analysis. This panel contains one row for each isolate in the phylogeny, with a column for each base in the reference genome. The background colour of each row represents the recipient cluster to which the isolate belongs. Along each row, changes of colour indicate putative recombinations; the colours indicate which of the donor clusters is most likely to have been the origin of the sequence. The overall pattern of recombination is similar to that observed in Figure 1, with little evidence of the import of sequence by clade IC1. As it is difficult to infer the directionality of exchange in some cases using BRATNextGen, recombinations observed in Figure 1 are sometimes reconstructed occurring in the same set of sequences, if both methods agree on which alleles are ancestral and which derived, but in other cases reconstructed as occurring in the complementary set of isolates, if the two methods disagree on which allele replaces which. These expected differences account for many of the superficial discrepancies between this analysis and that in Figure 1. Click here for file

Additional file 3: Figure S2 Distribution of homologous recombination lengths. Histograms showing the lengths of recombinations outside of MGEs for the entire collection (A) or just those recombinations within the PMEN2 clade (B). The red curves represent the fitting of exponential distributions, with the rate parameters 1.14 × 10^-4^ bp^-1^ (95% confidence interval, 1.04 × 10^-4^ to 1.26 × 10^-4^ bp^-1^) and 1.25 × 10^-4^ bp^-1^ (95% confidence interval, 1.11 × 10^-4^ to 1.42 × 10^-4^ bp^-1^), respectively. Click here for file

Additional file 4: Figure S3 Detail of the maximum likelihood phylogeny. The tree is coloured as in Figure 1, and the PMEN2 and PMEN22 clades labeled. All isolate names, as listed in Additional file 1: Table S1, are annotated on leaf nodes, while internal nodes are marked with the level of inferred support from 100 bootstrap replicates. Click here for file

Additional file 5: Figure S4 Distribution of Tn*916*-associated sequences. (A) Maximum likelihood phylogeny, as displayed in Figure 1. (B) Sequence of the Tn*916*-type element found in PMEN1, concatenated to the three macrolide resistance elements found in that lineage: the Omega, Tn*917* and mega cassettes. (C) Heatmap showing the mapping of Illumina reads to the sequences; blue indicates an absence of mapping, while red indicates a high level of mapping, with the maximum level capped at a depth of 10 fold coverage. Each row corresponds to one of the leaf nodes in the tree. Click here for file

Additional file 6: Figure S5 Distribution of Tn*5253*-type ICEs. (A) Maximum likelihood phylogeny, as displayed in Figure 1. (B) Distribution of ICE*Sp*6BST90. The annotated sequence of ICE*Sp*6BST90 is displayed across the top of the column, and a heatmap used to indicate the distribution of the ICE sequences across the collection, as described in Additional file 5: Figure S4. This demonstrates that ICE*Sp*6BST90 is found throughout PMEN2, although with sporadic deletion of the Ω*cat*(pC194)-like element. (C) Distribution of ICE*Sp*6BST273, displayed as described in (B). In this case, the full length element appears to be stable across the PMEN22 clade only. Click here for file

Additional file 7: Figure S6 Chloramphenicol resistance cassettes. The Ω*cat*(pC194) cassette previously identified as a mobile element in Tn*5253* is shown compared to the very similar element found within the PMEN22 isolate SPN13633 and the more divergent gene cluster present in ICE*Sp*6BST90. Red bands indicate regions of sequence similarity as identified by BLAT. The linearized plasmid pC194 is conserved in all the elements, as are the flanking sequences at the edges that include the 85 bp imperfect direct repeats. Click here for file

Additional file 8: Figure S7 Deletion of a section of Tn*916*-type ICE. Alignment of the Tn*916*-type component of ICE*Sp*6BST90 with the Tn*916*-type components of isolates SPN8332 (from this study), 11930 and 9409 (from the PMEN1 lineage). Red bands indicate regions of sequence similarity identified by BLAT. ICE*Sp*SPN8332 has been modified such that the majority of the Tn*916*-type component, including the *tetM* tetracycline resistance gene, has been replaced with an Omega cassette-type sequence, encoding an *aph3’* aminoglycoside phosphotransferase, and a Tn*917*-type sequence, encoding an *ermB* rRNA methylase. Each copy of the *ermB* gene is highlighted by an orange box. The generation of ICE*Sp*SPN8332 is likely to be the consequence of Omega and Tn*917* macrolide resistance cassettes both inserting into a Tn*916*-type element, as can be seen in ICE*Spn*11930 (although in this case, only a fragment of the Omega cassette remains; the full version is evident in ICE*Spn*9409). This results in two *ermB* genes arranged in tandem, between which an intragenomic recombination may occur that eliminates the intervening sequence. This results in the isolate being sensitive to tetracycline but resistant to aminoglycosides and macrolides. Click here for file

Additional file 9: Figure S8 Root-to-tip distance plots. (A) For each isolate within the PMEN2 clade associated with a precise year of isolation, this value was plotted against the distance of the corresponding sample from the root of the clade. This revealed a significant positive correlation (n = 168, *R*^2^ = 0.63, *P* 1;168, *R*^2^ = 0.63, 2.2 × 10^-16^) that provides evidence for a molecular clock signal in the data. (B) This shows the equivalent plot for the IC1 clade, where the date of isolation is plotted in terms of days, rather than years, again providing evidence of a molecular clock (n = 112, *R*^2^ = 0.56, *P* <2.2 × 10^-16^). Click here for file

Additional file 10: Figure S9 Distribution of prophage ΦIC1 sequence. (A) Maximum likelihood phylogeny, as displayed in Figure 1. (B) Annotation of prophage ΦIC1. The pink boxes represent CDSs, with their vertical position indicating whether they are encoded on the forward or reverse strand of the genome. The functional modules of the prophage are marked by the black bars across the top of the figure. The orange and brown bars indicate the extent of the scaffolds on which the two fragments of the phage are present, with the junction between them indicating a break in the draft assembly. (C) Heatmap showing the mapping of Illumina reads to the prophage sequence; blue indicates an absence of mapping, while red indicates high levels of mapping, with the maximum coverage level capped at 25 fold. Each row corresponds to one of the samples in the tree. Isolates of clade IC1 tended to show consistent mapping across the entire sequence if the prophage is present in their genome; many other samples only show mapping to parts of the sequence owing to the presence of related prophage. Mapping to the lysogeny module, which contains the integrase that determines the insertion site of the virus, is indicative of prophage inserted into the *comYC* gene. Click here for file

Additional file 11: Figure S10 Prophage inserting into *comYC*. As isolates were frequently polylysogenic, many prophage were difficult to assemble. These four examples represent the most complete assemblies of prophage inserting into the *comYC* gene. The top sequence is taken from the complete reference genome; prophage ΦIC1 spans two scaffolds, with the assembly break between them indicated by the vertical dashed line; the sequences of ΦDCC1738 and ΦK13-0810 are present on a single contig, but the lytic amidase is truncated by an assembly break in both. The pink boxes mark CDSs, with their vertical position indicating whether they are encoded on the forward or reverse strand of the genome. The functional modules of the prophage are marked by the black bars across the top of the figure. The red bands between sequences indicate BLAT matches, with the intensity of the colour representing the strength of the match. It can be seen that the integrase within the lysogeny module is conserved, reflecting the common insertion site shared by the elements. Click here for file

Additional file 12: Table S2 Comparison of different evolutionary models fitted to the PMEN2 clade using BEAST. Five different models are compared using log_10_ Bayes factors. Positive values of Bayes factors indicates a comparatively better fit of the model described in the row to the data relative to the model indicated by the column heading. Click here for file

Additional file 13: Table S3 Comparison of different evolutionary models fitted to the IC1 clade using BEAST. Five different models are compared using log_10_ Bayes factors. Positive values of Bayes factors indicates a comparatively better fit of the model described in the row to the data relative to the model indicated by the column heading. Click here for file
