# Supplementary figures and images for: Variable recombination dynamics during the emergence, transmission and ‘disarming’ of a multidrug-resistant pneumococcal clone
Source: BMC Biol. 2014 Jun 23;12:49. doi: 10.1186/1741-7007-12-49 (PMC4094930; doi:10.1186/1741-7007-12-49)

Figure S1

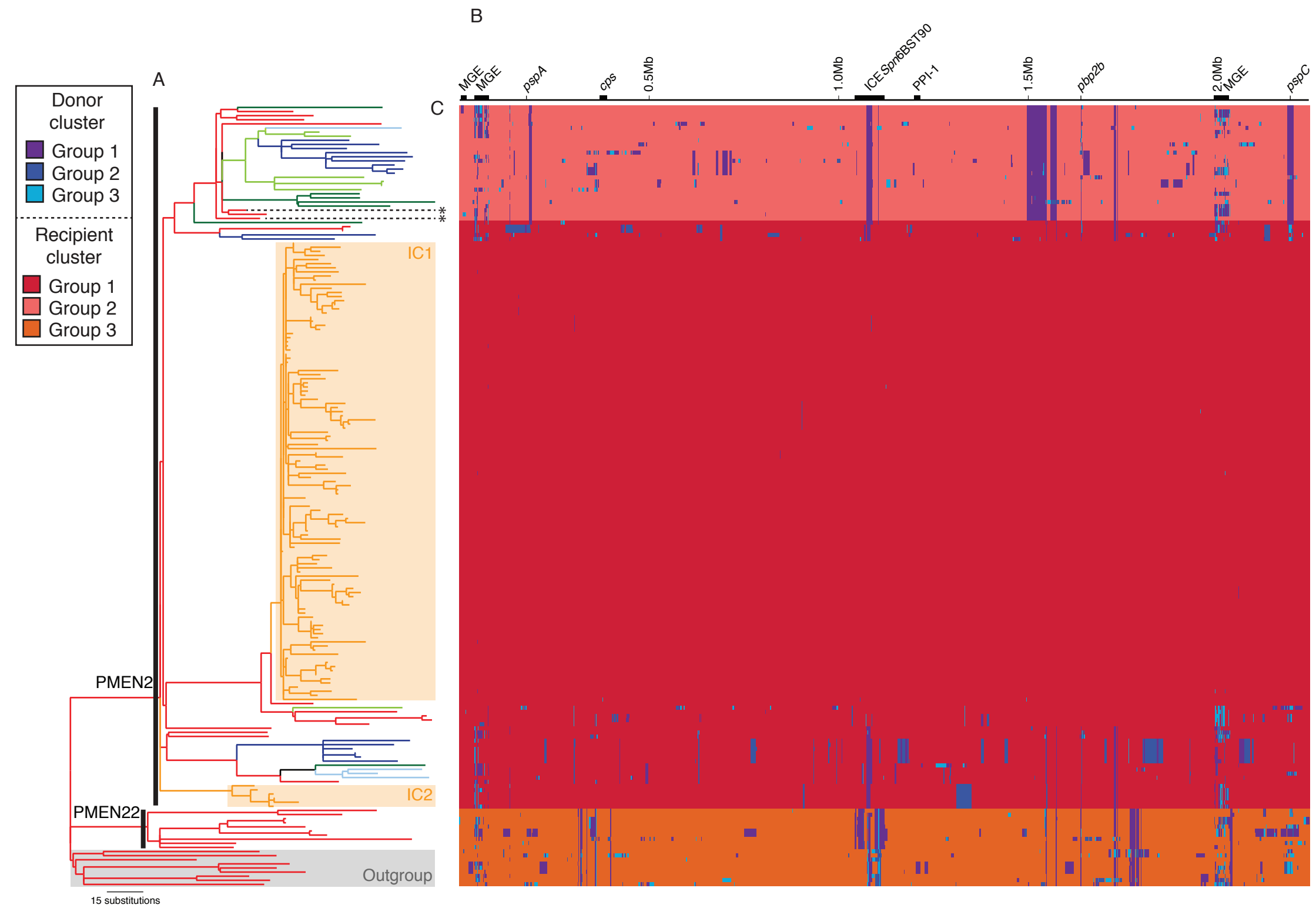

Supplement: Additional file 2: Figure S1 — Analysis of the whole genome alignment using BRATNextGen. (A) Maximum likelihood phylogeny, as displayed in Figure 1. (B) Simplified annotation of the reference genome as displayed in Figure 1. (C) Results of the BRATNextGen analysis. This panel contains one row for each isolate in the phylogeny, with a column for each base in the reference genome. The background colour of each row represents the recipient cluster to which the isolate belongs. Along each row, changes of colour indicate putative recombinations; the colours indicate which of the donor clusters is most likely to have been the origin of the sequence. The overall pattern of recombination is similar to that observed in Figure 1, with little evidence of the import of sequence by clade IC1. As it is difficult to infer the directionality of exchange in some cases using BRATNextGen, recombinations observed in Figure 1 are sometimes reconstructed occurring in the same set of sequences, if both methods agree on which alleles are ancestral and which derived, but in other cases reconstructed as occurring in the complementary set of isolates, if the two methods disagree on which allele replaces which. These expected differences account for many of the superficial discrepancies between this analysis and that in Figure 1. [file 1741-7007-12-49-S2.pdf]

Figure S2

A

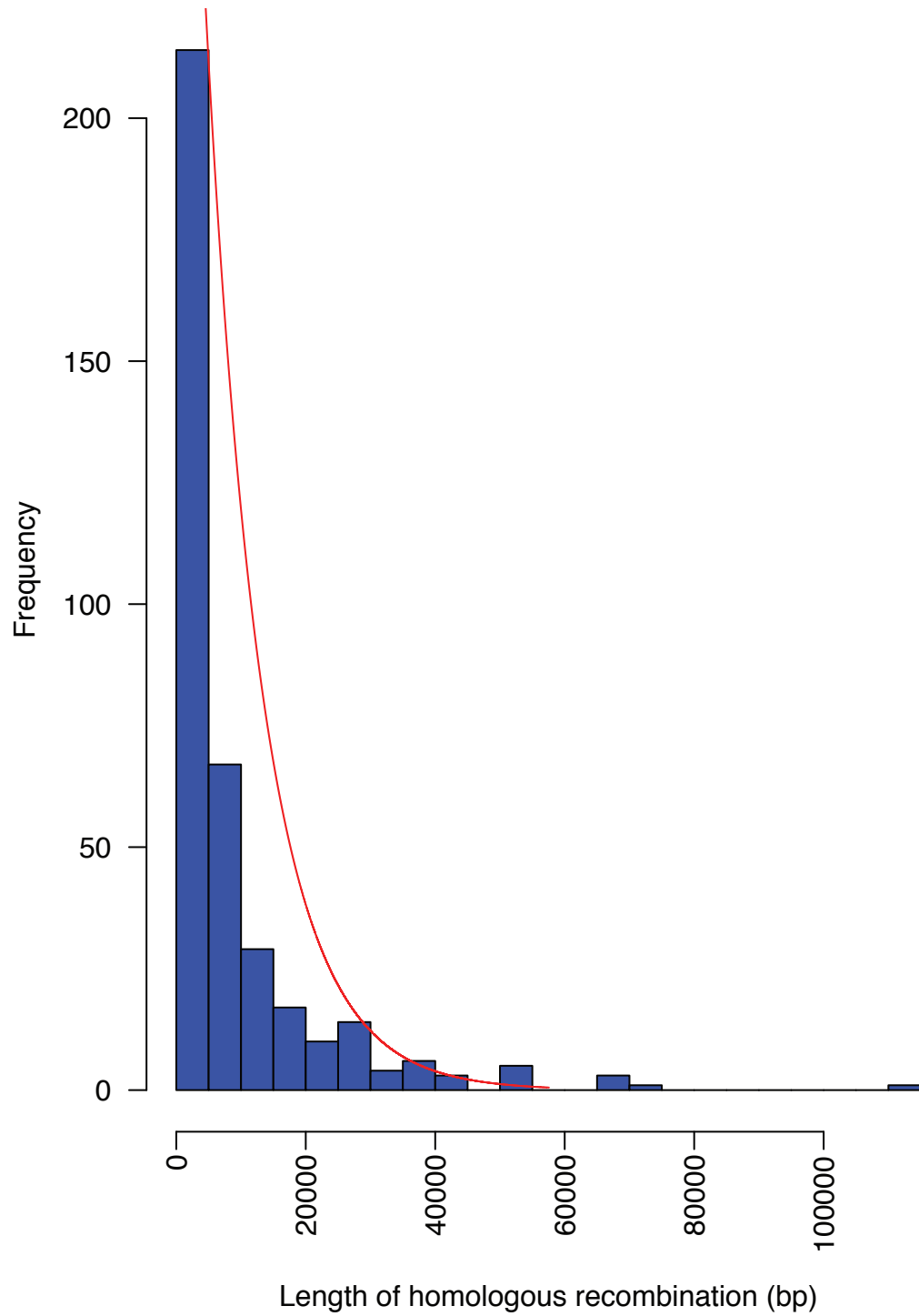

B

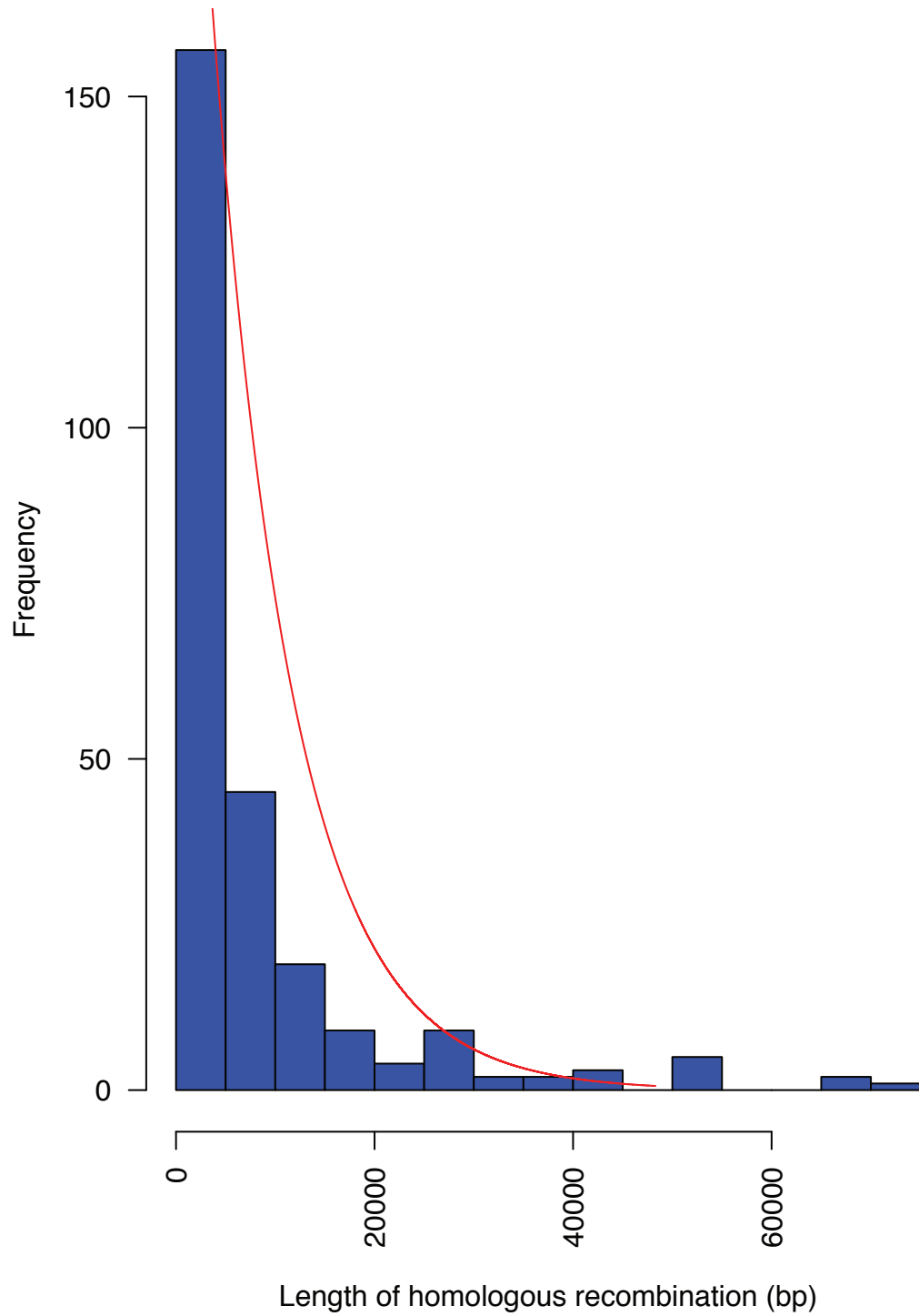

Supplement: Additional file 3: Figure S2 — Distribution of homologous recombination lengths. Histograms showing the lengths of recombinations outside of MGEs for the entire collection (A) or just those recombinations within the PMEN2 clade (B). The red curves represent the fitting of exponential distributions, with the rate parameters 1.14 × 10-4 bp-1 (95% confidence interval, 1.04 × 10^-4^ to 1.26 × 10-4 bp-1) and 1.25 × 10-4 bp-1 (95% confidence interval, 1.11 × 10^-4^ to 1.42 × 10-4 bp-1), respectively. [file 1741-7007-12-49-S3.pdf]

PMEN22

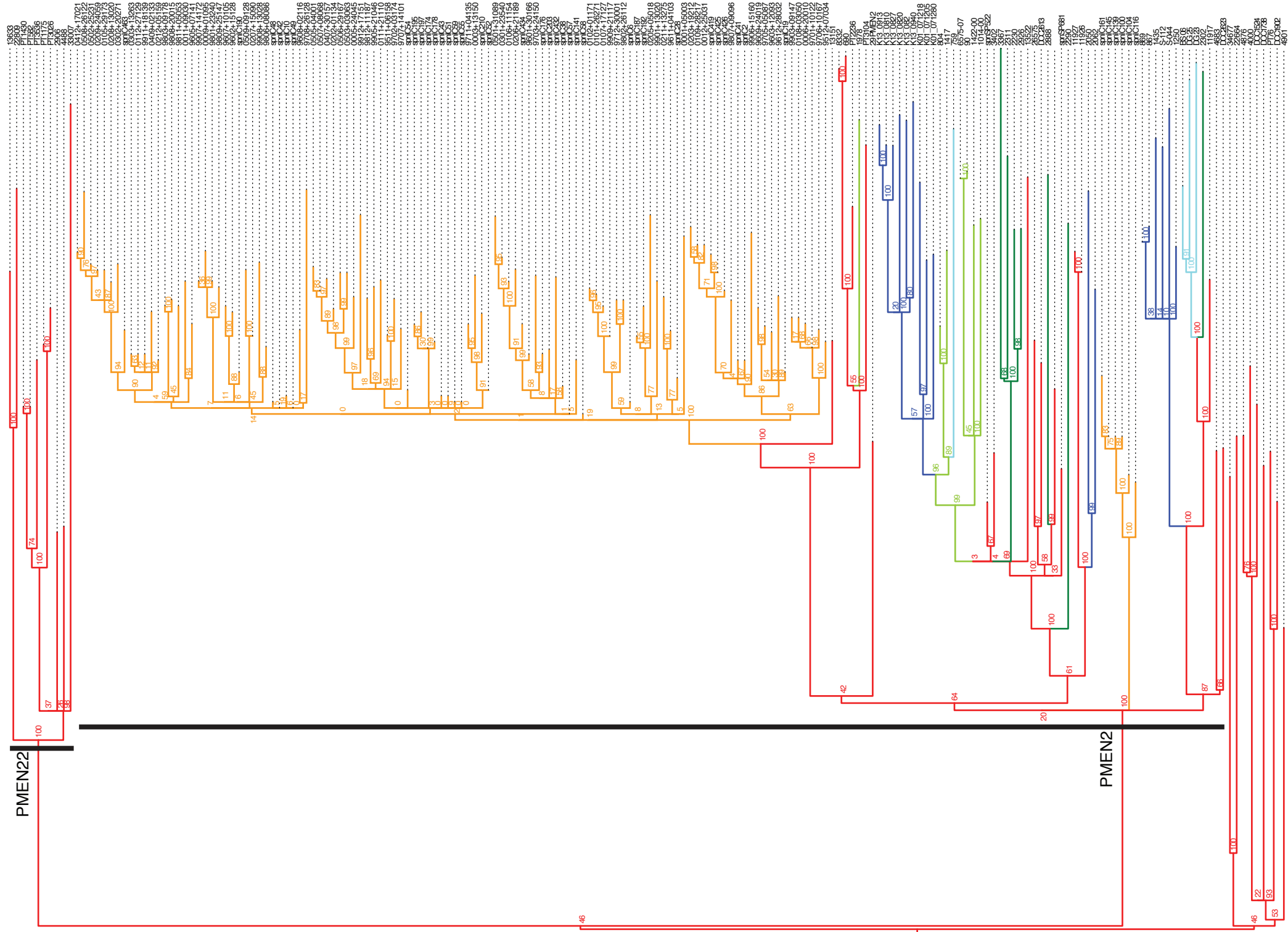

## 24.7 substitutions

Supplement: Additional file 4: Figure S3 — Detail of the maximum likelihood phylogeny. The tree is coloured as in Figure 1, and the PMEN2 and PMEN22 clades labeled. All isolate names, as listed in Additional file 1: Table S1, are annotated on leaf nodes, while internal nodes are marked with the level of inferred support from 100 bootstrap replicates. [file 1741-7007-12-49-S4.pdf]

Figure S4

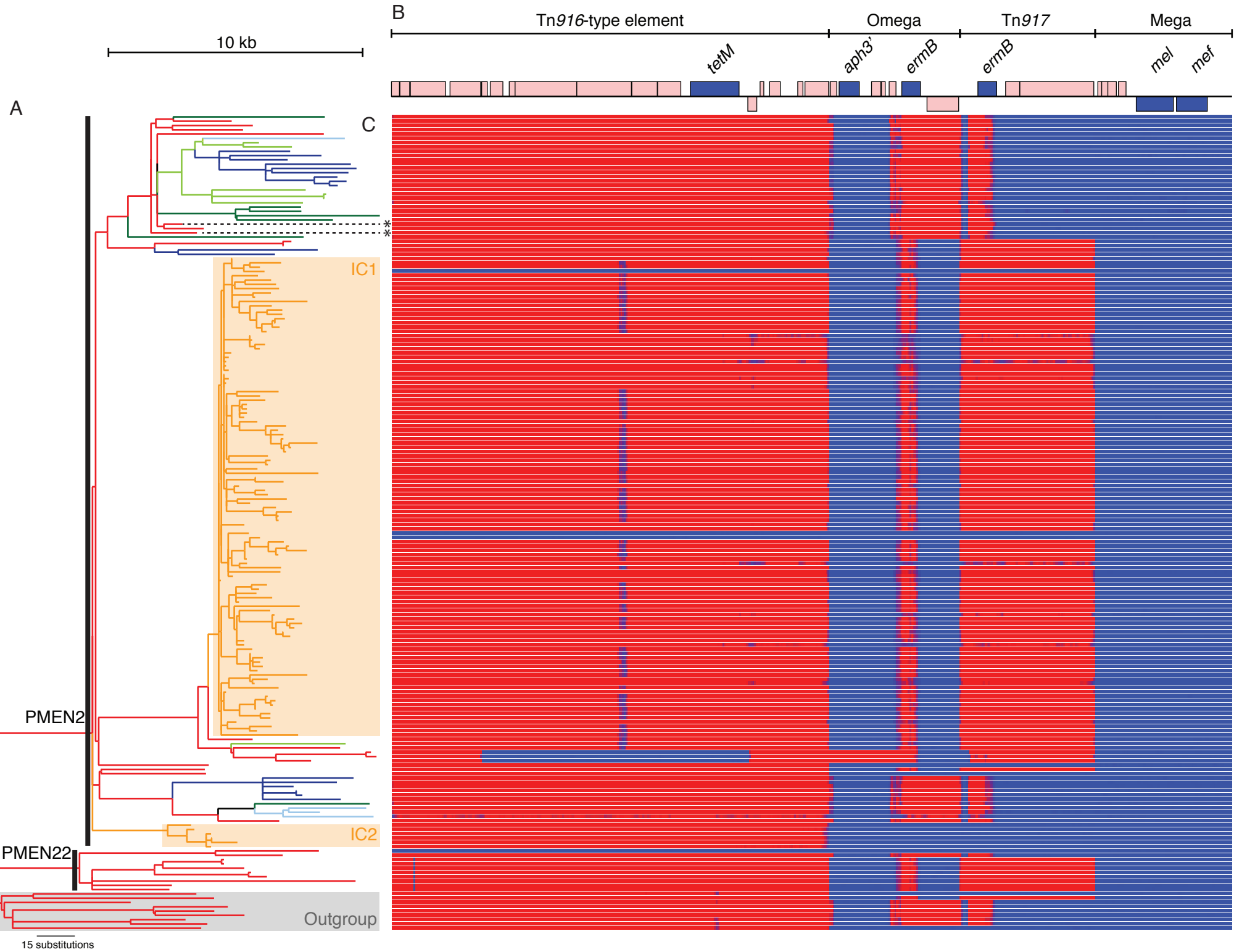

Supplement: Additional file 5: Figure S4 — Distribution of Tn916-associated sequences. (A) Maximum likelihood phylogeny, as displayed in Figure 1. (B) Sequence of the Tn916-type element found in PMEN1, concatenated to the three macrolide resistance elements found in that lineage: the Omega, Tn917 and mega cassettes. (C) Heatmap showing the mapping of Illumina reads to the sequences; blue indicates an absence of mapping, while red indicates a high level of mapping, with the maximum level capped at a depth of 10 fold coverage. Each row corresponds to one of the leaf nodes in the tree. [file 1741-7007-12-49-S5.pdf]

Figure S5

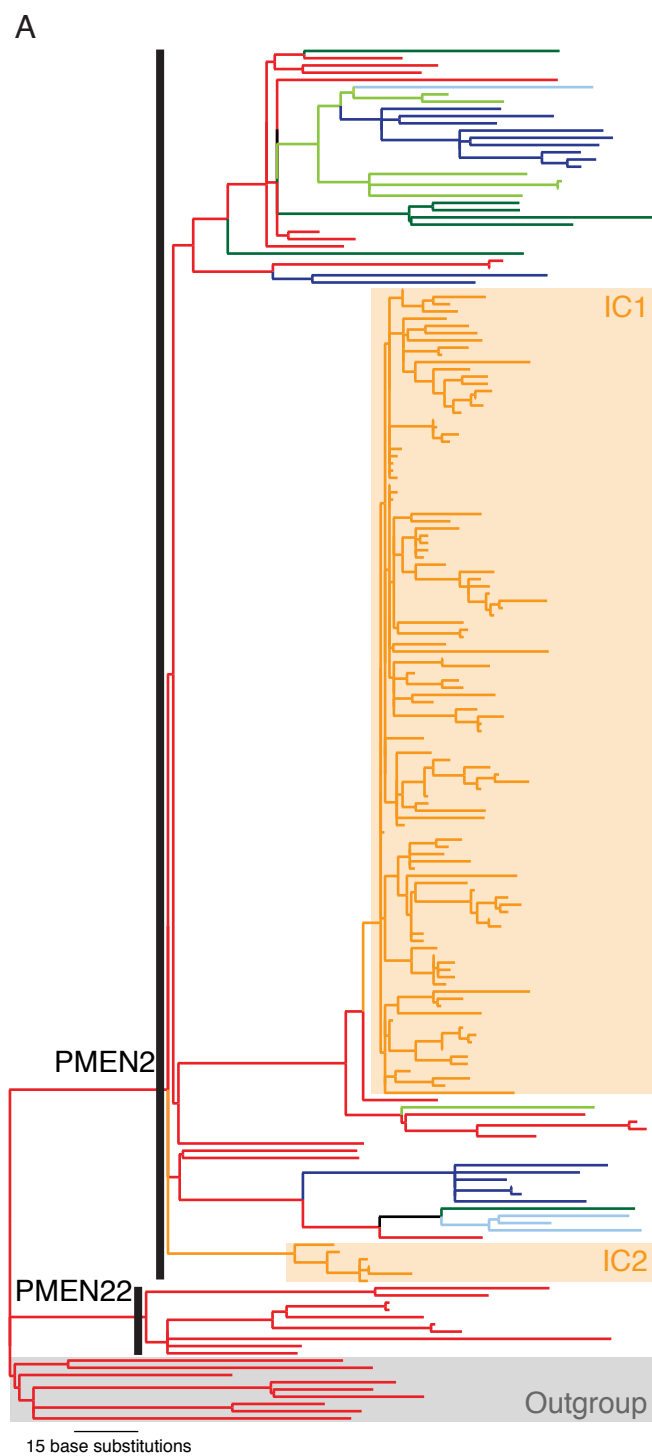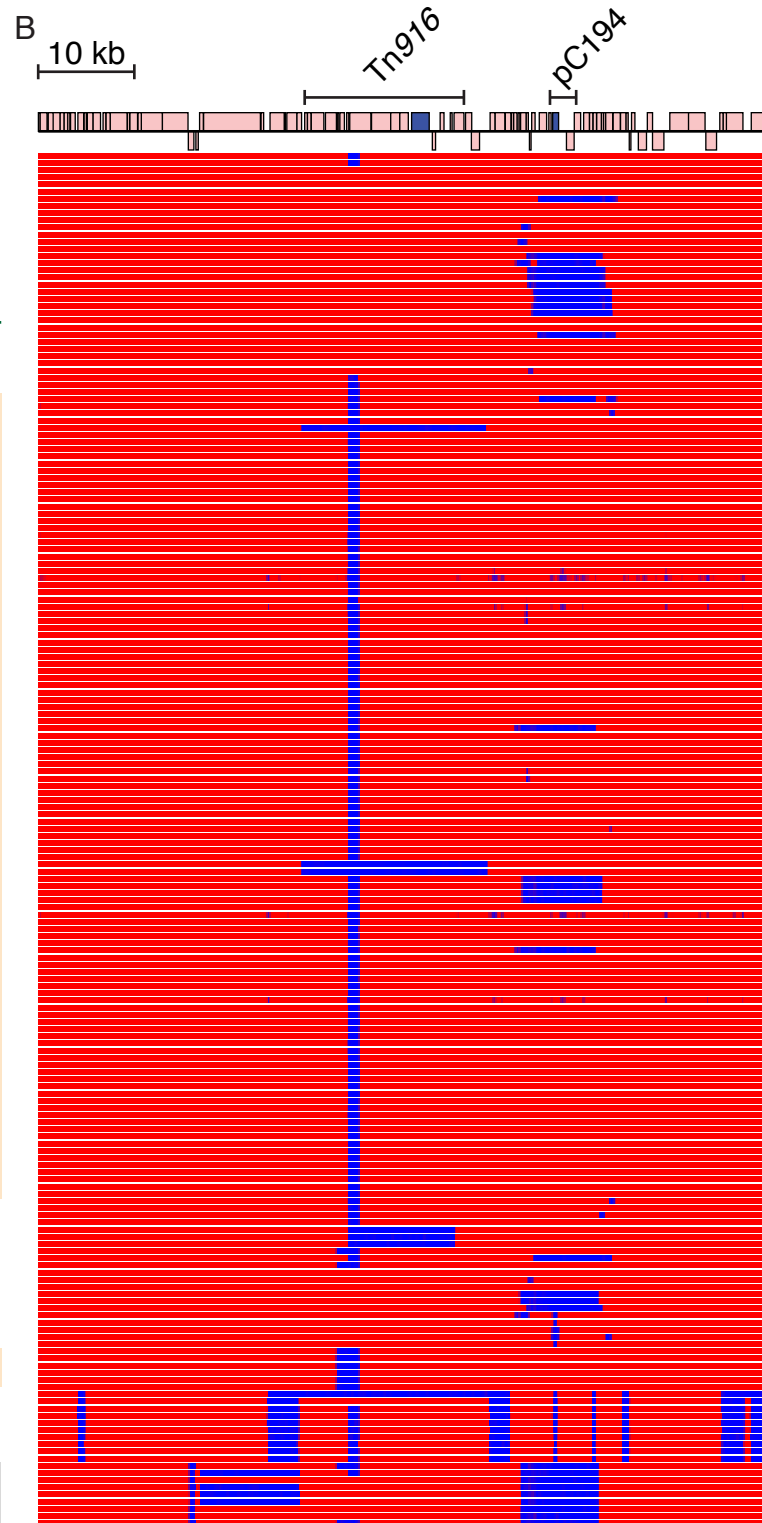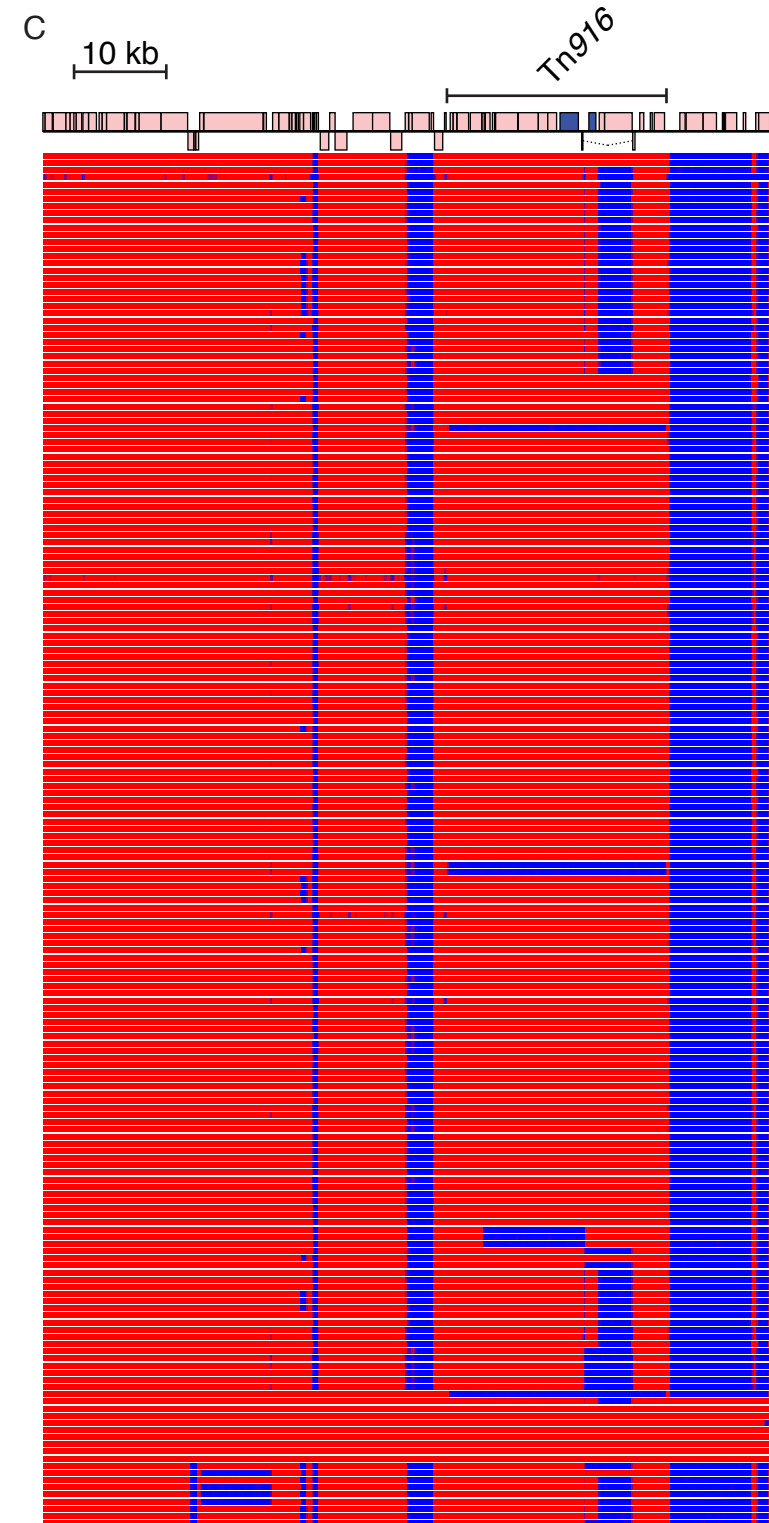

Supplement: Additional file 6: Figure S5 — Distribution of Tn5253-type ICEs. (A) Maximum likelihood phylogeny, as displayed in Figure 1. (B) Distribution of ICESp6BST90. The annotated sequence of ICESp6BST90 is displayed across the top of the column, and a heatmap used to indicate the distribution of the ICE sequences across the collection, as described in Additional file 5: Figure S4. This demonstrates that ICESp6BST90 is found throughout PMEN2, although with sporadic deletion of the Ωcat(pC194)-like element. (C) Distribution of ICESp6BST273, displayed as described in (B). In this case, the full length element appears to be stable across the PMEN22 clade only. [file 1741-7007-12-49-S6.pdf]

Figure S6

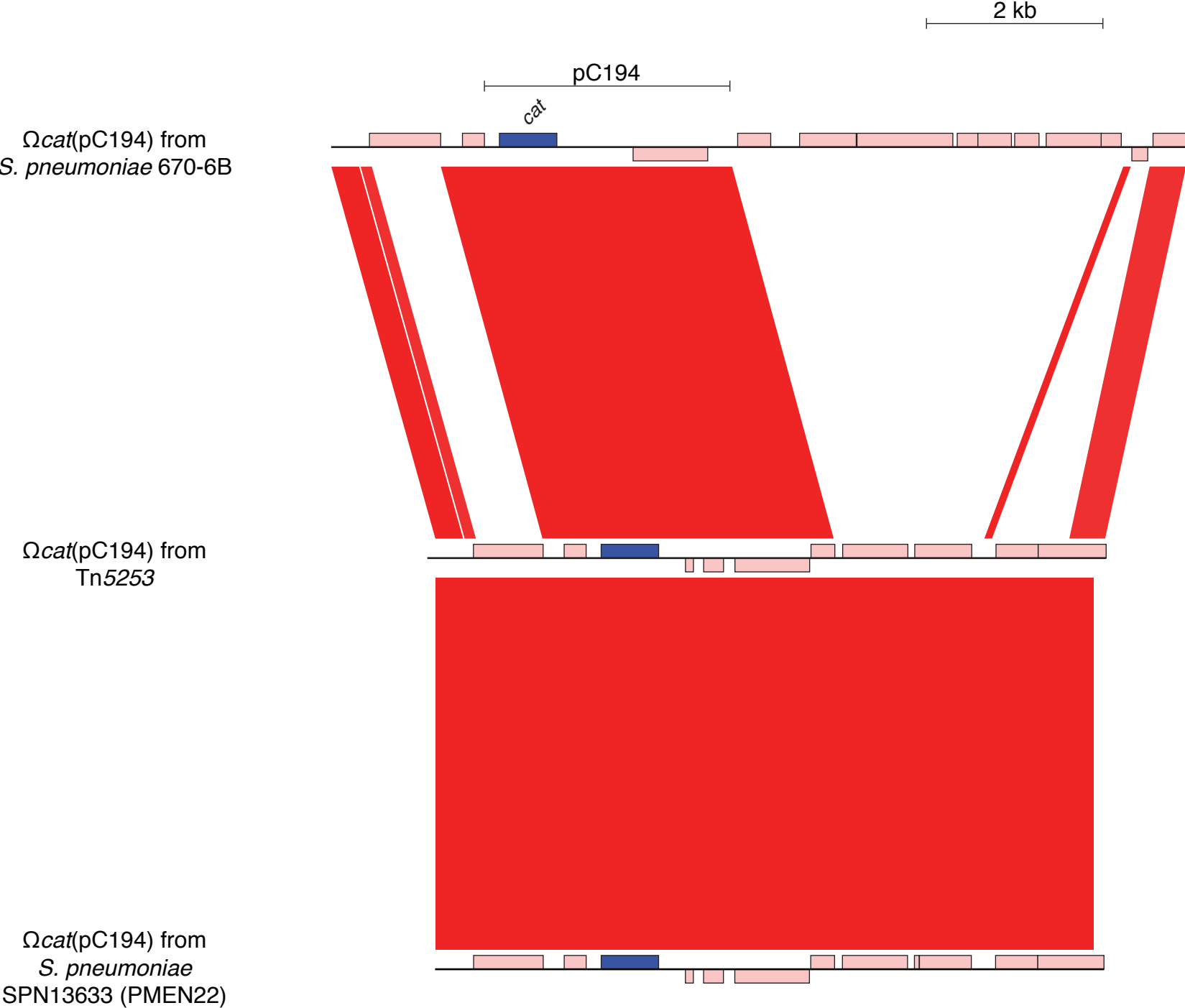

Supplement: Additional file 7: Figure S6 — Chloramphenicol resistance cassettes. The Ωcat(pC194) cassette previously identified as a mobile element in Tn5253 is shown compared to the very similar element found within the PMEN22 isolate SPN13633 and the more divergent gene cluster present in ICESp6BST90. Red bands indicate regions of sequence similarity as identified by BLAT. The linearized plasmid pC194 is conserved in all the elements, as are the flanking sequences at the edges that include the 85 bp imperfect direct repeats. [file 1741-7007-12-49-S7.pdf]

Figure S7

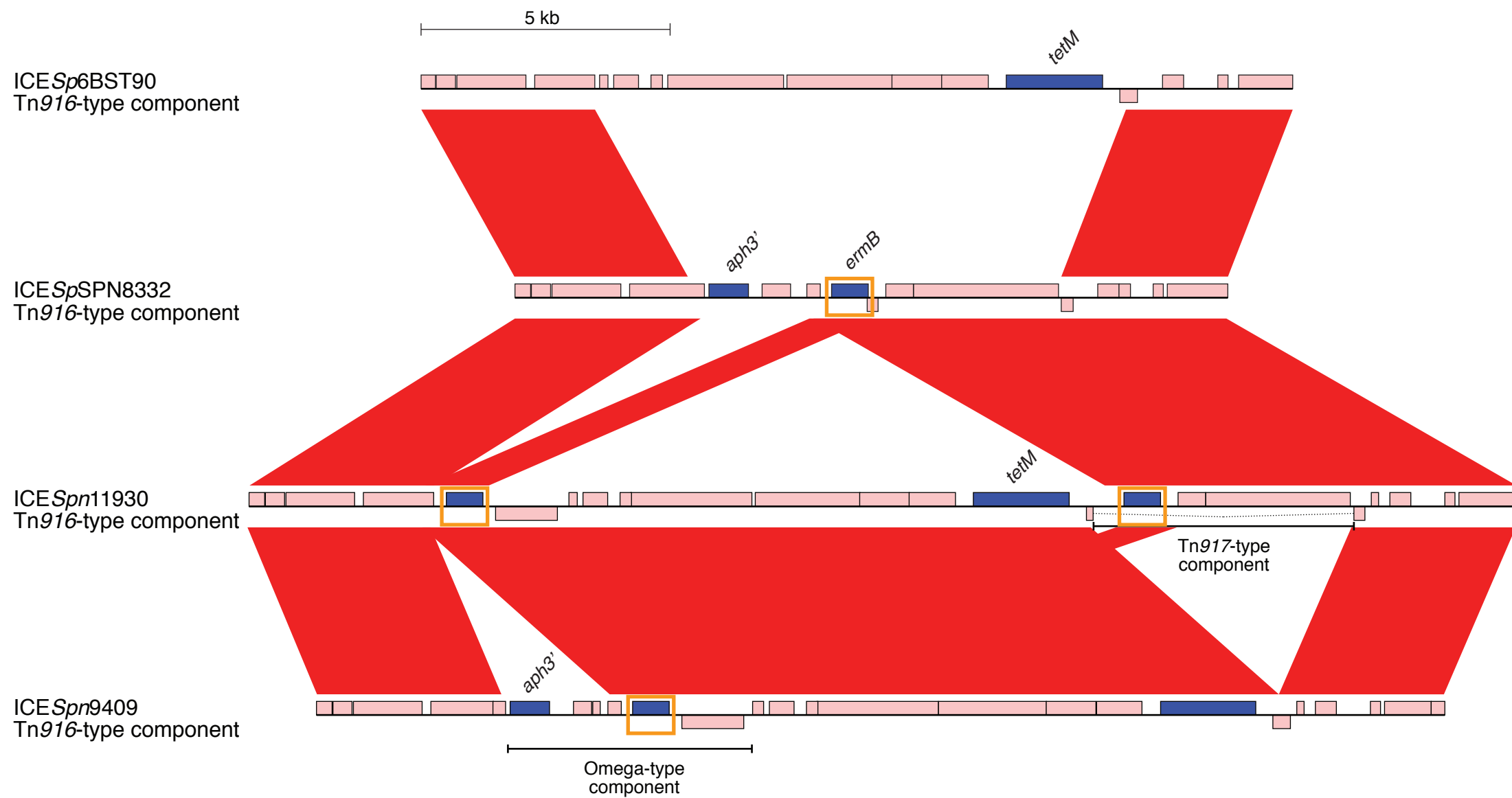

Supplement: Additional file 8: Figure S7 — Deletion of a section of Tn916-type ICE. Alignment of the Tn916-type component of ICESp6BST90 with the Tn916-type components of isolates SPN8332 (from this study), 11930 and 9409 (from the PMEN1 lineage). Red bands indicate regions of sequence similarity identified by BLAT. ICESpSPN8332 has been modified such that the majority of the Tn916-type component, including the tetM tetracycline resistance gene, has been replaced with an Omega cassette-type sequence, encoding an aph3’ aminoglycoside phosphotransferase, and a Tn917-type sequence, encoding an ermB rRNA methylase. Each copy of the ermB gene is highlighted by an orange box. The generation of ICESpSPN8332 is likely to be the consequence of Omega and Tn917 macrolide resistance cassettes both inserting into a Tn916-type element, as can be seen in ICESpn11930 (although in this case, only a fragment of the Omega cassette remains; the full version is evident in ICESpn9409). This results in two ermB genes arranged in tandem, between which an intragenomic recombination may occur that eliminates the intervening sequence. This results in the isolate being sensitive to tetracycline but resistant to aminoglycosides and macrolides. [file 1741-7007-12-49-S8.pdf]

Figure S8

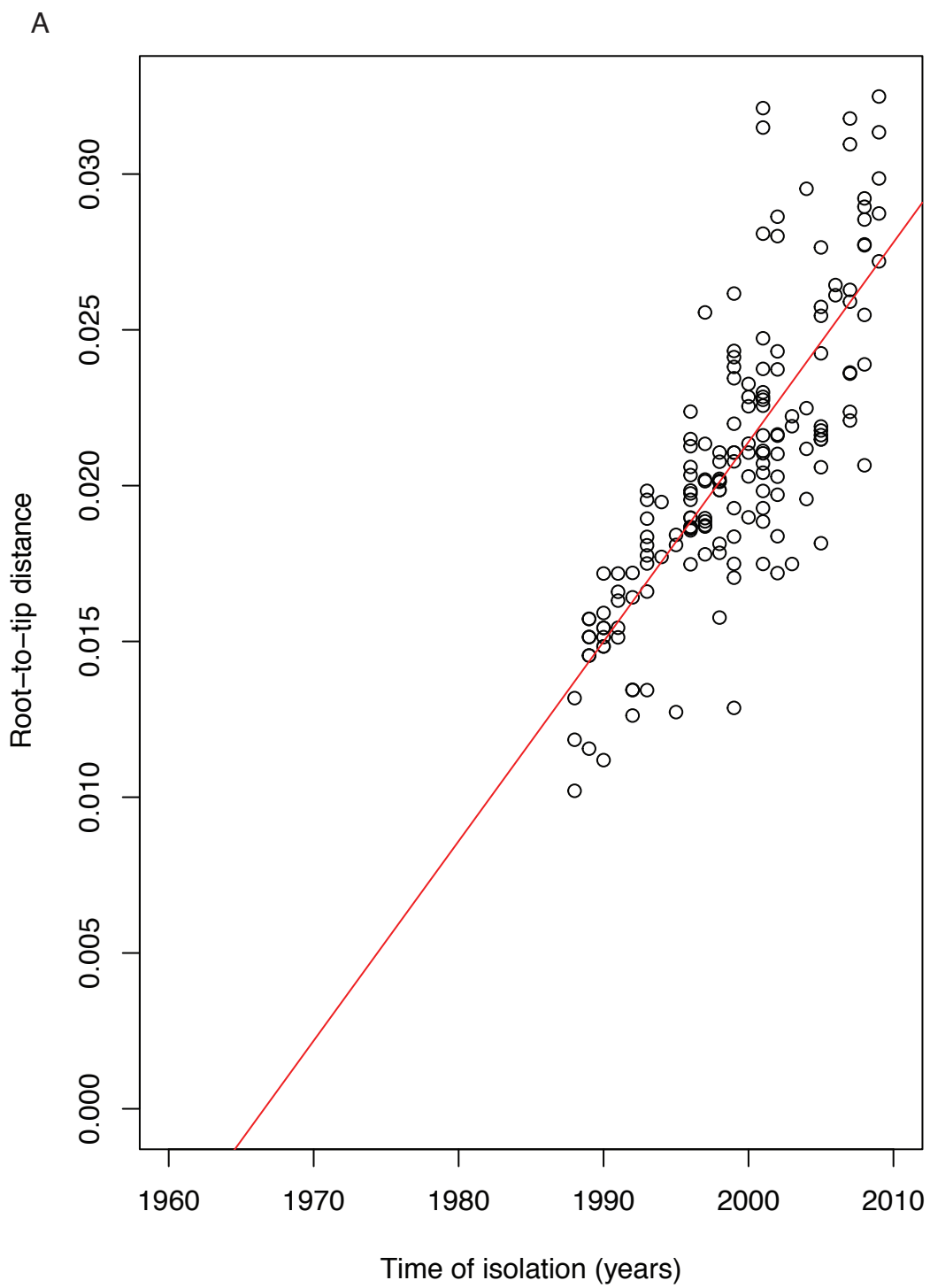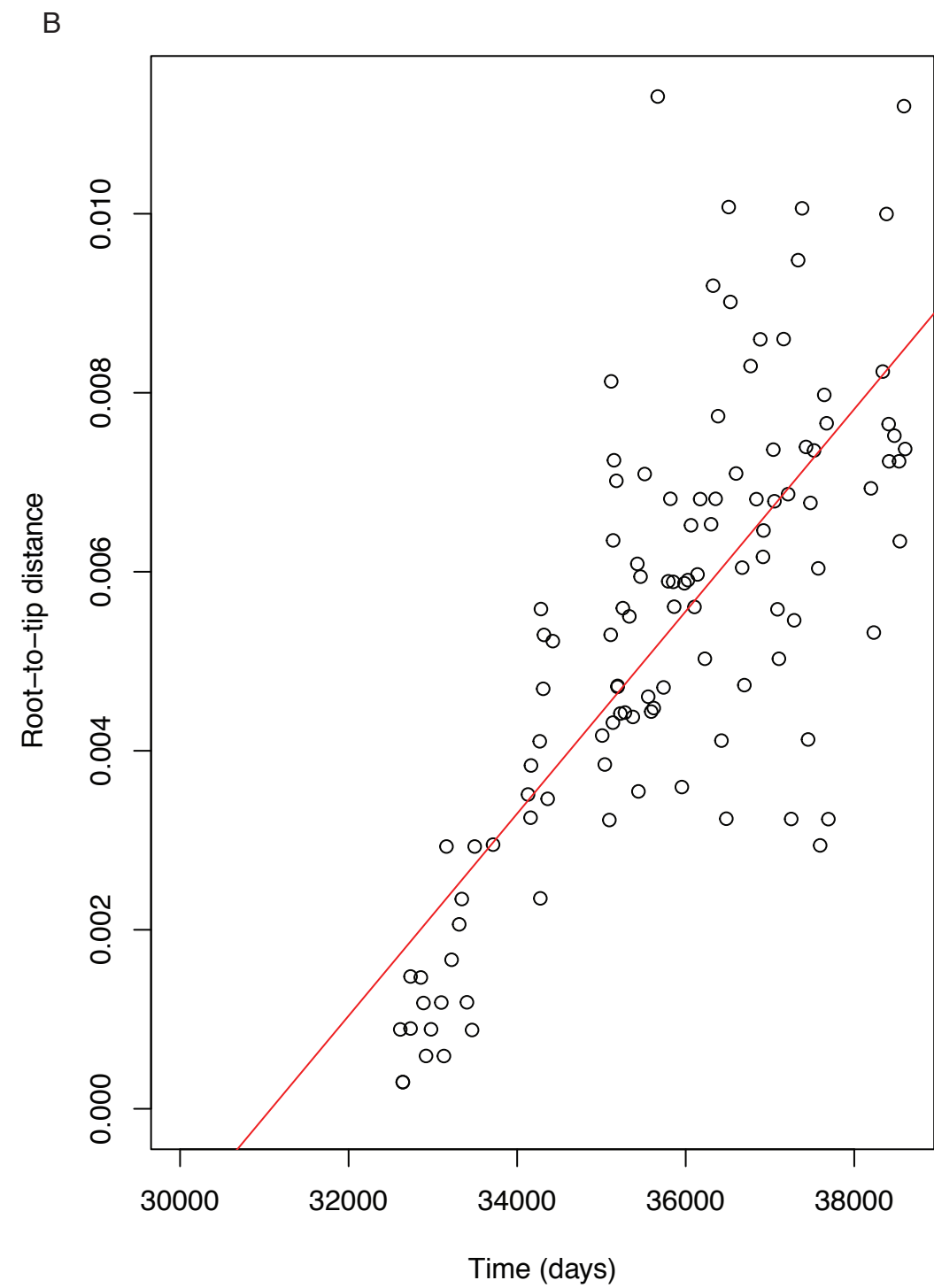

Supplement: Additional file 9: Figure S8 — Root-to-tip distance plots. (A) For each isolate within the PMEN2 clade associated with a precise year of isolation, this value was plotted against the distance of the corresponding sample from the root of the clade. This revealed a significant positive correlation (n = 168, R2 = 0.63, P 1;168, R2 = 0.63, 2.2 × 10^-16^) that provides evidence for a molecular clock signal in the data. (B) This shows the equivalent plot for the IC1 clade, where the date of isolation is plotted in terms of days, rather than years, again providing evidence of a molecular clock (n = 112, R2 = 0.56, P <2.2 × 10^-16^). [file 1741-7007-12-49-S9.pdf]

Figure S9

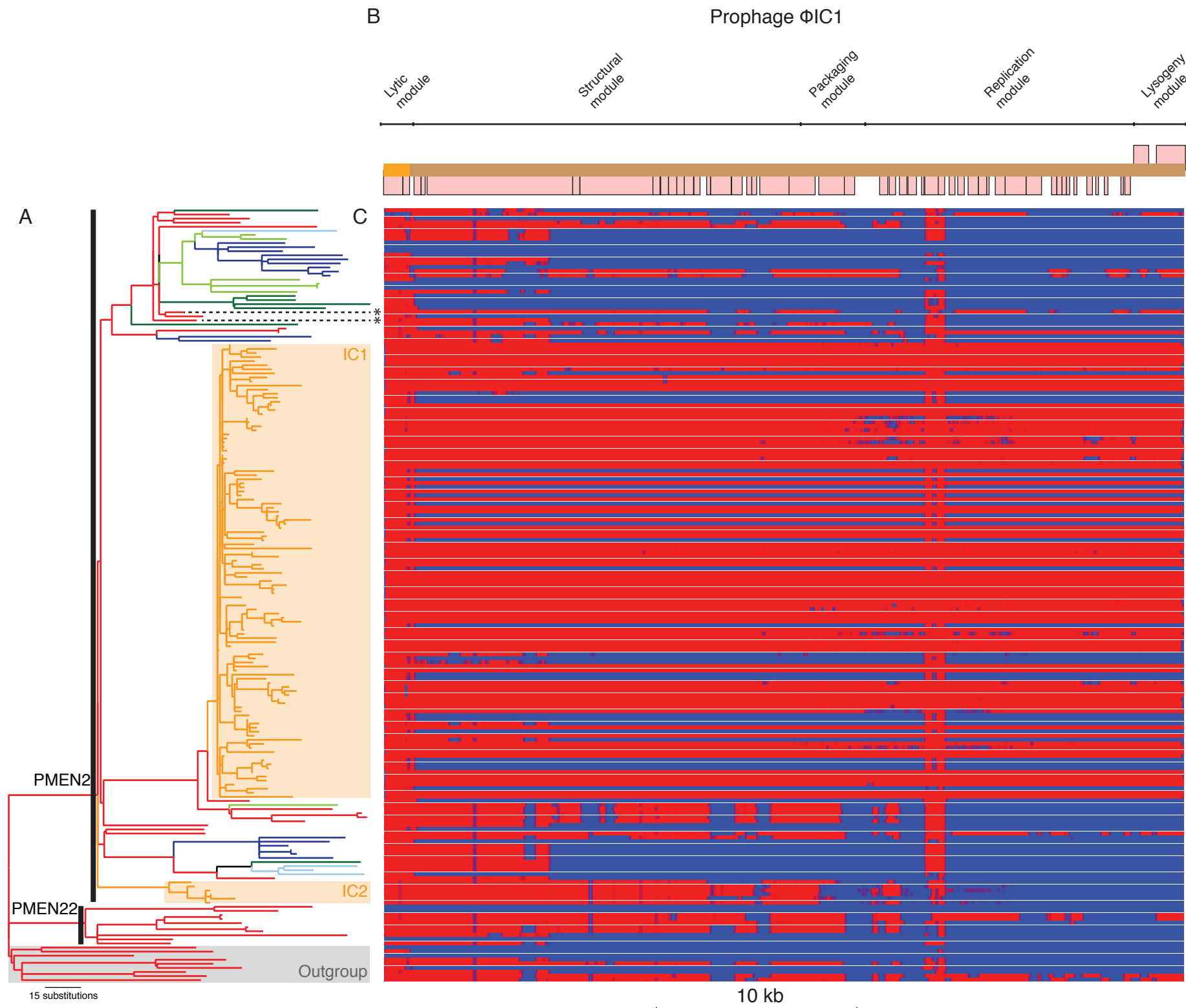

Supplement: Additional file 10: Figure S9 — Distribution of prophage ΦIC1 sequence. (A) Maximum likelihood phylogeny, as displayed in Figure 1. (B) Annotation of prophage ΦIC1. The pink boxes represent CDSs, with their vertical position indicating whether they are encoded on the forward or reverse strand of the genome. The functional modules of the prophage are marked by the black bars across the top of the figure. The orange and brown bars indicate the extent of the scaffolds on which the two fragments of the phage are present, with the junction between them indicating a break in the draft assembly. (C) Heatmap showing the mapping of Illumina reads to the prophage sequence; blue indicates an absence of mapping, while red indicates high levels of mapping, with the maximum coverage level capped at 25 fold. Each row corresponds to one of the samples in the tree. Isolates of clade IC1 tended to show consistent mapping across the entire sequence if the prophage is present in their genome; many other samples only show mapping to parts of the sequence owing to the presence of related prophage. Mapping to the lysogeny module, which contains the integrase that determines the insertion site of the virus, is indicative of prophage inserted into the comYC gene. [file 1741-7007-12-49-S10.pdf]

Figure S10

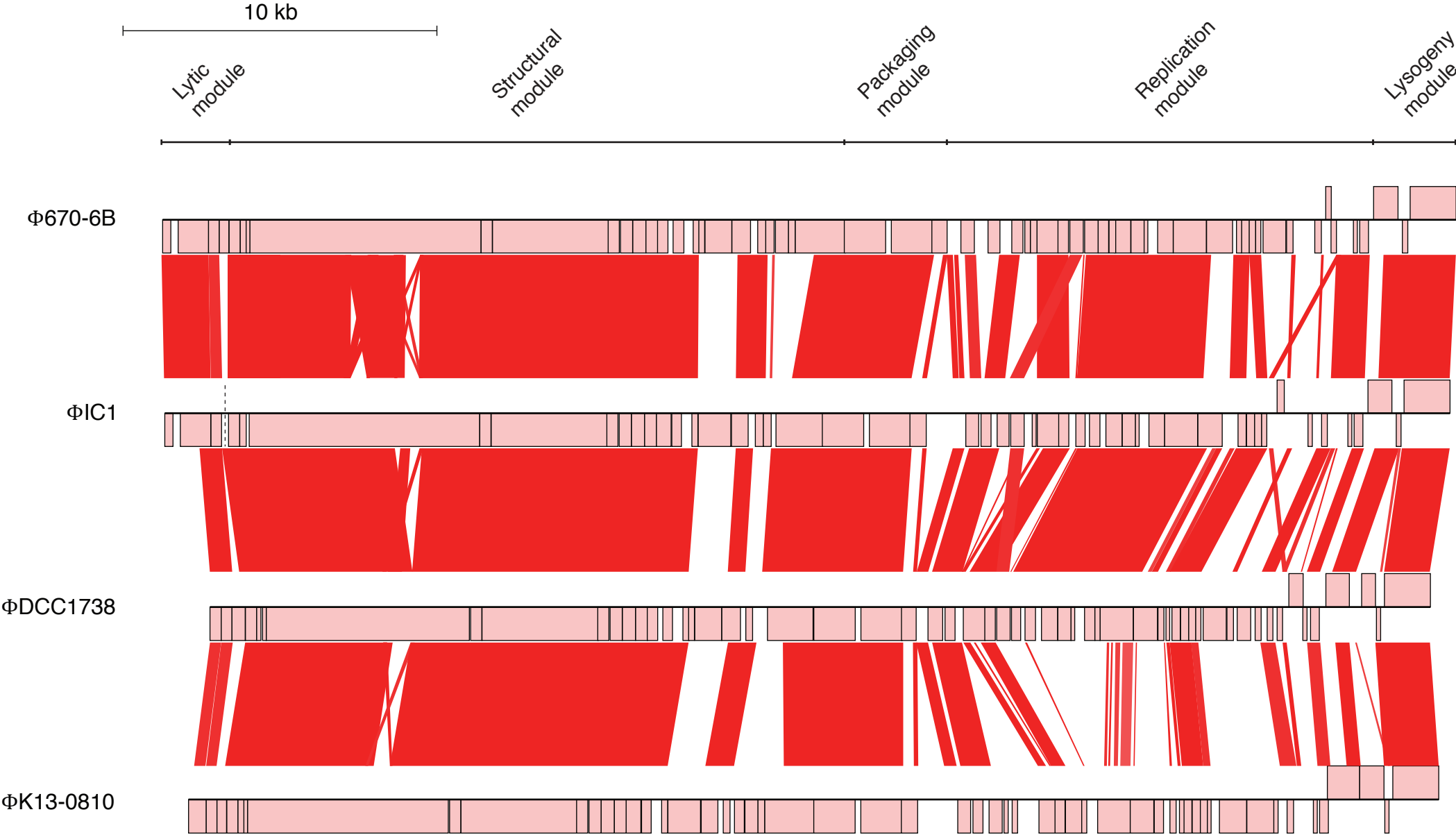

Supplement: Additional file 11: Figure S10 — Prophage inserting into comYC. As isolates were frequently polylysogenic, many prophage were difficult to assemble. These four examples represent the most complete assemblies of prophage inserting into the comYC gene. The top sequence is taken from the complete reference genome; prophage ΦIC1 spans two scaffolds, with the assembly break between them indicated by the vertical dashed line; the sequences of ΦDCC1738 and ΦK13-0810 are present on a single contig, but the lytic amidase is truncated by an assembly break in both. The pink boxes mark CDSs, with their vertical position indicating whether they are encoded on the forward or reverse strand of the genome. The functional modules of the prophage are marked by the black bars across the top of the figure. The red bands between sequences indicate BLAT matches, with the intensity of the colour representing the strength of the match. It can be seen that the integrase within the lysogeny module is conserved, reflecting the common insertion site shared by the elements. [file 1741-7007-12-49-S11.pdf]
