# Supplementary material for: Variable recombination dynamics during the emergence, transmission and ‘disarming’ of a multidrug-resistant pneumococcal clone
Source: BMC Biol. 2014 Jun 23;12:49. doi: 10.1186/1741-7007-12-49 (PMC4094930; doi:10.1186/1741-7007-12-49)
Supplement: Additional file 12: Table S2 — Comparison of different evolutionary models fitted to the PMEN2 clade using BEAST. Five different models are compared using log10 Bayes factors. Positive values of Bayes factors indicates a comparatively better fit of the model described in the row to the data relative to the model indicated by the column heading. [file 1741-7007-12-49-S12.docx]

| **Model Number** | **Substitution Model** | **Clock Model** | **Number of rate categories** | **ln P(model\|data)** | **SE** | **Comparison against other models**  **(log_10_ Bayes Factor)** | | | | |
| --- | --- | --- | --- | --- | --- | --- | --- | --- | --- | --- |
|  |  |  |  |  |  | **1** | **2** | **3** | **4** | **5** |
| 1 | GTR | Strict | 1 | -22945 | 0.057 | - | -11.7 | -41.7 | -41.3 | 40.5 |
| 2 | GTR | Random | 1 | -22918 | 0.061 | 11.7 | - | -29.9 | -29.5 | 52.3 |
| 3 | GTR | Relaxed lognormal | 1 | -22849 | 0.066 | 41.7 | 29.9 | - | 0.39 | 82.2 |
| 4 | GTR | Relaxed lognormal | 4 | -22850 | 0.068 | 41.3 | 29.5 | -0.39 | - | 81.8 |
| 5 | HKY | Relaxed lognormal | 1 | -23039 | 0.066 | -40.5 | -52.3 | -82.2 | -81.8 | - |
