# Supplementary material for: Variable recombination dynamics during the emergence, transmission and ‘disarming’ of a multidrug-resistant pneumococcal clone
Source: BMC Biol. 2014 Jun 23;12:49. doi: 10.1186/1741-7007-12-49 (PMC4094930; doi:10.1186/1741-7007-12-49)
Supplement: Additional file 13: Table S3 — Comparison of different evolutionary models fitted to the IC1 clade using BEAST. Five different models are compared using log10 Bayes factors. Positive values of Bayes factors indicates a comparatively better fit of the model described in the row to the data relative to the model indicated by the column heading. [file 1741-7007-12-49-S13.docx]

| **Model Number** | **Substitution Model** | **Clock Model** | **Number of rate categories** | **ln P(model\|data)** | **SE** | **Comparison against other models**  **(log_10_ Bayes Factor)** | | | | |
| --- | --- | --- | --- | --- | --- | --- | --- | --- | --- | --- |
|  |  |  |  |  |  | **1** | **2** | **3** | **4** | **5** |
| 1 | GTR | Strict | 1 | -7650 | 0.272 | - | -5.13 | -13.2 | -13.9 | 25.7 |
| 2 | GTR | Random | 1 | -7638 | 0.321 | 5.13 | - | -8.03 | -8.77 | 30.8 |
| 3 | GTR | Relaxed lognormal | 1 | -7620 | 0.442 | 13.2 | 8.03 | - | -0.743 | 38.8 |
| 4 | GTR | Relaxed lognormal | 4 | -7618 | 0.358 | 13.9 | 8.77 | 0.743 | - | 39.6 |
| 5 | HKY | Relaxed lognormal | 1 | -7709 | 0.337 | -25.7 | -30.8 | -38.8 | -39.6 | - |
